# A machine learning approach to discover migration modes and transition dynamics of heterogeneous dendritic cells

**DOI:** 10.3389/fimmu.2023.1129600

**Published:** 2023-04-04

**Authors:** Taegeun Song, Yongjun Choi, Jae-Hyung Jeon, Yoon-Kyoung Cho

**Affiliations:** ^1^ Department of Physics, Pohang University of Science and Technology (POSTECH), Pohang, Republic of Korea; ^2^ Department of Data information and Physics, Kongju National University, Gongju, Republic of Korea; ^3^ Center for Soft and Living Matter, Institute for Basic Science (IBS), Ulsan, Republic of Korea; ^4^ Department of Biomedical Engineering, Ulsan National Institute of Science and Technology (UNIST), Ulsan, Republic of Korea; ^5^ Asia Pacific Center for Theoretical Physics (APCTP), Pohang, Republic of Korea

**Keywords:** dendritic cell, cell migration, machine learning, transition dynamics, maturation

## Abstract

Dendritic cell (DC) migration is crucial for mounting immune responses. Immature DCs (imDCs) reportedly sense infections, while mature DCs (mDCs) move quickly to lymph nodes to deliver antigens to T cells. However, their highly heterogeneous and complex innate motility remains elusive. Here, we used an unsupervised machine learning (ML) approach to analyze long-term, two-dimensional migration trajectories of Granulocyte-macrophage colony-stimulating factor (GMCSF)-derived bone marrow-derived DCs (BMDCs). We discovered three migratory modes independent of the cell state: slow-diffusive (SD), slow-persistent (SP), and fast-persistent (FP). Remarkably, imDCs more frequently changed their modes, predominantly following a unicyclic SD→FP→SP→SD transition, whereas mDCs showed no transition directionality. We report that DC migration exhibits a history-dependent mode transition and maturation-dependent motility changes are emergent properties of the dynamic switching of the three migratory modes. Our ML-based investigation provides new insights into studying complex cellular migratory behavior.

## 1 Introduction

Cell migration is essential for homeostasis in living systems (1). Intriguingly, cell motility shows complex dynamics beyond the classical diffusion theory (2). Therefore, various random-walk models have been employed to explain anomalous diffusion processes (3, 4). For instance, bacterial micro-swimmers and T cells deploy an effective intermittent search process, alternating between slow and fast motion, such as the run-and-tumble motion and Lévy walk (5, 6).

Dendritic cells (DCs) exhibit adaptive motility patterns, reflecting their immunological function as major antigen-presenting cells (7, 8). Immature DCs (imDCs) typically navigate using an intermittent search strategy, combining fast persistent motility during patrolling and slow-diffusive motility for antigen collection (9–11). In contrast, mature DCs (mDCs) mainly employ fast persistent motility, enabling them to reach lymph nodes and deliver antigens to T cells (12, 13). A recent single-cell study employing microfabricated devices showed that deterministic actin waves contribute to the intermittent search mechanism (14). Additionally, identified Lévy walk patterns showed directional persistence and zigzag motion as an *in vivo* search strategy (15). Although extensive studies have been performed on the overall cellular migration characteristics and physiological framework of DC motility, the description of the average migration dynamics is mostly restricted to the dichotomous approach, such as slow or fast, diffusive or persistent, zigzag or non-zigzag.

We hypothesized that such simple interpretations overlook the complex and heterogeneous dynamics of single-cell motility. Intrigued by experimental observations of the heterogeneous distribution of DC motility (**Supplementary Figure S1**), we designed an unsupervised machine learning (ML) method to uncover DC motility patterns at the single-cell level and understand the distinct dynamic modes and their transition dynamics quantitatively.

ML analysis is a powerful tool for classifying complex natural phenomena. In the field of single-particle trajectory analysis (16), ML has been exploited to reuse imperfect datasets (17), retrieve information on location or polarization (18), and infer transport models (19–21). ML technique was also applied to classifying various mathematical diffusion models and cell mobility patterns using trajectory data (22, 23). They differentiated the trajectory-to-trajectory variation based on user-defined features. In addition, deep-learning approaches have been proposed to extract characteristic features from trajectories (24–26).

In this study, we developed an ML method to quantitatively analyze complex and heterogeneous cellular motility processes and discovered that the DC migratory patterns were classified into three distinctive modes with unique characteristics. Our ML is a hybrid machine combining unsupervised learning with supervised learning. Compared to the above ML tools classifying cell-to-cell variation patterns, our ML method was adapted to analyzing temporally heterogeneous cell migration motion. Moreover, our ML algorithm used only a minimal number of features specific to characterizing cell migration motion and is interpretable in terms of feature importance. We investigated the distribution and dynamic transitions between these three modes and found that motility changes upon maturation are emergent properties of these processes. This ML-enabled approach paves the way for the subsequent investigation of complex cellular migration dynamics.

## 2 Materials and methods

### 2.1 GM-CSF-derived BMDCs

BMDCs were generated as previously described (12, 13). Briefly, tibias and femurs from BALB/c mice (8–12 weeks old, female) were flushed, and red blood cells were lysed with ammonium-chloride-potassium (ACK) lysis buffer (Gibco). Bone marrow cells were plated in 24 well tissue culture well plates (1 × 10^6^ cells/mL) in complete medium containing RPMI 1640, supplemented with 5% fetal bovine serum (FBS), 1% antibiotic-antimycotic solution, 1% HEPES buffer, and 0.1% 2-mercaptoethanol (all reagents were purchased from Gibco) containing 20 ng/mL recombinant mouse granulocyte-macrophage colony-stimulating factor (GM-CSF; Peprotech). The medium was completely replaced with fresh GM-CSF every two days. On day six, non-adherent and loosely adherent cells were collected by gentle pipetting and transferred to Petri dishes. After one day of culture, immature BMDCs, which appeared as floating cells, were collected. Phenol-red-free RPMI 1640 medium (Gibco) was used for fluorescence microscopy experiments, including cell height measurements and cell viability tests. To generate mature dendritic cells (mDCs), immature DCs (imDCs) were stimulated with 100 ng/mL lipopolysaccharide (LPS, LPS-EB Ultrapure; Invivogen) for 30 min. Cells were carefully washed three times and incubated for 6 h in fresh complete medium, as described in previous studies (12, 13). After incubation, floating cells were harvested as mDCs. All animal experiments were performed according to protocols approved by the Institutional Animal Care and Use Committee of the Ulsan National Institute of Science and Technology (UNISTIACUC-19-15).

### 2.2 DC characterization

The specific surface markers of imDCs and mDCs were characterized by flow cytometry (Cytoflex, Beckman Coulter; **Supplementary Figure S2**). The upregulated expression levels of co-stimulatory molecules, CD86, CD80, and CD40, antigen-presenting molecule MHC class II (I-A/I-E), and the chemokine receptor CCR7 were evaluated as DC maturation markers, and CD11c and CD11b were analyzed as dendritic cell markers. The following antibodies were used in flow cytometry experiments: anti-CD86-FITC (GL1, 1:200), anti-CD40-PE (1C10, 1:200), anti-MHC class II (I-A/I-E)-FITC (M5/114.15.2, 1:200), and anti-CCR7-PE (4B12, 1:200) were purchased from Thermo Fisher Scientific, and anti-CD11b-APC (M1/70, 1:200), anti-CD11c-APC (HL3, 1:200), and anti-CD80-PE (16-10A1, 1:200) were purchased from BD Biosciences. The acquired data were analyzed using the FlowJo software (BD).

### 2.3 Fabrication of gel confiner

Under-agarose migration by gel confinement was performed as described previously (13, 27). Briefly, the gel confiner consisted of a custom-designed PDMS structure and low-melting agarose gel. The PDMS structure consisted of a 10:1 ratio for the main body and a 30:1 ratio for the sticky PDMS-coated bottom. Before casting the gel solution, the PDMS structure was placed in a Petri dish. Low-melting agarose (2.4%) was dissolved in phenol-red free HBSS buffer and heated at 80°C for 20 min, and the resulting solution was cooled at room temperature to 40°C. The same volume of 2× conditioned medium (RPMI 1640, 10% FBS, 2% HEPES buffer, 2% antibiotic-antimycotic solution, and 0.2% 2-mercaptoethanol) was mixed to a final concentration of 1.2% and cast to the PDMS structure. The gel was cured for 20 min at room temperature. The cured gel confinement was incubated overnight in a cell culture incubator with complete medium. Subsequently, to prevent non-specific cell-to-cell interactions in the migration assay, 800 cells in a small drop of cell suspension were seeded on 10 mm diameter coverslips with 20 μg/mL bovine fibronectin-coating. The cells were incubated for 30 min in a cell culture incubator to enable them to settle on the substrate. Subsequently, the cells were carefully covered by gel confinement, and motility was imaged after 1 h. In the experiments to measure cell motility, the field of view (FOV) was located within the radial distance of 1.7 ± 0.6 mm from the center, and there was no significant difference in cell behavior observed at the center and edge positions.

### 2.4 Measurement of Young’s moduli of the agarose gel block

Agarose gel blocks were prepared at concentrations of 1.2% (w/v) with the same gel confinement fabrication. The mechanical properties of the gels were determined using a rheometer (MCR502 WESP; Anton Paar). The gel height was approximately 1.0 mm. The shear moduli (G, Pa) were analyzed using the relationship between shear stress and shear strain before gel disruption, and Young’s moduli (E, kPa) were calculated using the following equation: E = 2(1 + v)G, with the Poisson’s ratio (v) of agarose set as 0.5 (28).

### 2.5 Measurement of cell height under confinement

To check the reproducibility of the confined environment, the height of the fluorescence-stained DCs under 2D gel confinement was measured using a laser scanning confocal microscope (A1R, Nikon), as shown in **Supplementary Figure S3**. The DC suspensions were stained using DiO (Invitrogen) for three minutes and washed thrice with complete medium. After three minutes of recovery, stained DCs were seeded onto the substrate and covered by gel confinement. The 3D confocal image was acquired using a 100x plan apo lens, and the *z* interval was 0.5 μm. Cell height was measured manually using a NIS Element (Nikon).

### 2.6 Live-cell imaging

Live-cell imaging was performed using an inverted microscope (Eclipse Ti-E, Nikon) configured with a 10x dry objective lens and sCMOS camera (Flash4.0, Hamamatsu). The cells were imaged for 24 h, and bright-field images were obtained every 1 min. The recorded images were processed using the *z*-max intensity projection for non-labeled automatic tracking (29). The sample focal plane was focused, and two more image sequences were obtained along the *z*-axis. Subsequently, rolling-ball background subtraction was performed, and the maximum intensity projection overlaid the *z*-stacks, facilitating contrast enhancement for cell segmentation. During the experiments, an incubator (Chamlide HK; Live Cell Instrument) maintained the microscope at 37°C with 95% humidity and 5% CO_2_.

### 2.7 Tracking of cellular migration

For tracking, cells in the pre-processed image were automatically detected using the IMARIS (Bitplane) ‘Spots’ function. Cells were identified using intensity thresholding and size estimation as 20 μm diameter particles. The position of the center of mass was tracked, and tracking errors such as misconnections between different objects or misrecognized objects were corrected manually.

### 2.8 Data pre-processing

We obtained the following raw trajectories of DC migration:348 (imDCs, training dataset), 383 (mDCs, training dataset), 449 (imDCs, testing dataset), and 350 (mDCs, testing dataset). Among them, a few raw trajectories were not tracked perfectly, and as a result, a few data points were absent in the trajectories. Therefore, we polished such trajectories. The number of imperfect trajectories (fraction) was identified as 16 (4.5%, imDC training dataset), 20 (5.22%, mDCs training dataset), 26 (5.79%, imDC testing dataset), and 20 (5.71%, mDCs testing dataset). The missing data points were attributed to tracking errors that mainly arise from cell-to-cell interactions during attachment between two distinct cells and partially from the finite field of view, resulting in the incomplete observation of cell movement near the edge of the observation window. Thus, we manually analyzed the trajectories using the following rule: First, we chose an arbitrary time window (≤ 10 min) and applied the spline interpolation provided in the Python library “scipy” package to the raw data (30). If the untracked period was ≥10 min, we split the trajectory into two before and after attachment. Splitting an imperfectly tracked trajectory into two independent short trajectories does not seriously alter the statistics and the detection of migration patterns of single trajectories because this portion is approximately 4%–6% of the trajectory data. We also evaluated the interval of missing points, such as 20 min or 30 min time windows; however, the results shown in analyzed motility data did not differ qualitatively. If we consider three dynamical modes, the statistical properties of the modes are robust against the interval of missing points owing to the relatively few imperfect trajectories obtained.

After data polishing, we removed inappropriate track samples in our ML analysis that failed to satisfy any of the following three criteria as previously referred (12, 14): Mean track speed ( $<V_{D}{>}_{t}\geq 1.5\;\text{μm}/\text{min}$ ), track duration ( $T_{obs}\geq 60\;\text{min}$ ), and maximal displacement from the starting position ( $\max\left|\vec{R}\right|\geq 20\;\text{μm}$ ).

### 2.9 Hybird Machine learning kernel

We constructed a hybrid ML kernel combining two machines each used as unsupervised and supervised learning. Firstly, the K-means clustering was applied to generate pseudo labels for 1 h long segmented trajectories as a training data set. Then, randomly chosen 2000 trajectories (36.6%) are used for training XGBOOST. The trained XGBOOST predicted the label for unknown trajectories in the segment pool with a 99.6% accuracy.

In our novel kernel design, the classical K-means clustering with silhouette score systematically determines the number of clusters, and the decision-tree-based XGBOOST provides the importance of the feature. The benefits of two distinct types of machines are providing a deeper understanding of trajectories. Thus, our designed kernel possibly adapts to similar single-particle-tracking data analysis with user-defined features.

### 2.10 Silhouette score used to determine number of clusters

The silhouette score has been widely used to infer the number of clusters in ML algorithms (31). With the given cluster numbers as a hyperparameter, we measured the mean distance of a given data point within the cluster to which it belongs. For data point *i*, the mean distance within the cluster is estimated as $D_{W}\left(i\right)=\frac{1}{\left|C_{I}\right|}\sum_{j\in C_{I}}d\left(i,j\right),\;$ where d(i,j) represents the Euclidian distance between the *i* and j  data points in the cluster $C_{I},$ and $\left|C_{I}\right|$ is the size of the cluster. We estimated the dissimilarity of the data point *i* by measuring the minimum distance between the data point and other clusters, which is defined as $D_{D}\left(i\right)=\underset{\left\{C_{J}\right\}}{\min}\frac{1}{\left|C_{J}\right|}\sum_{j\in C_{J}}d\left(i,j\right),$ where $i\in C_{I},$ $j\in C_{J}$ , and $\left\{C_{J}\right\}$ is the set of all neighboring clusters for J≠I. The silhouette score for data point i is defined as $s\left(i\right)=\frac{D_{D}\left(i\right)-D_{w}\left(i\right)}{\max\left[D_{w}\left(i\right),D_{D}\left(i\right)\right]}$ . Thus, the average silhouette score lies between [−1,1] . The limit of 1 describes a sample (i.e., cluster) that is well separated from neighboring clusters.

### 2.11 Molecular inhibition in the migration assay

To study the role of myosin II and the Arp2/3 complex, the molecular inhibitors blebbistatin (20 μM, Sigma-Aldrich), CK666 (100 μM, Sigma-Aldrich) and SMIFH2 (12.5 μM, Sigma-Aldrich) were used during DC migration. Each inhibitor was mixed in an uncured gel (40°C) before casting. After gel casting and curing at RT, the gel confiner was incubated in complete medium with the same inhibitor concentration overnight. DMSO (0.1% (v/v); Sigma-Aldrich) was used as a negative control. Subsequently, the DCs located on the substrate were carefully covered with an inhibitor-containing gel confiner. Cell motility assays were conducted after 1 h of incubation with covering by the gel confiner, similar to the approach described above.

### 2.12 DC viability under inhibitor effect

Ethidium homodimer (EthD-1; Thermo Fisher Scientific) was used to evaluate DC viability. After 24 h of incubation, DCs under gel confinement were stained with EthD-1 in complete medium (500 nM) in an incubator for 20 min. Fluorescent-stained dead cells were counted manually using epifluorescence microscopy with a 20× Plan Apo lens (Nikon TiE). Cell survival rate was calculated as the number of stained cells per the number of whole cells in the field of view. More than 50 cells were analyzed for each experiment, and three independent experiments were performed.

### 2.13 Statistical analysis

Unless stated otherwise, all data represent the mean ± SEM of three independent experiments for each condition. Normality was determined using the D’Agostino and Pearson tests. The Mann-Whitney and Kruskal-Wallis tests, along with Dunn’s *post hoc* test, were used to determine statistical significance. To prevent type I errors due to the large number of samples and confirm statistical significance, randomly selected samples were used to obtain the *P*-values. The criteria for random selection followed the hypothesized sample size, with a statistical power of 0.8. All statistical analyses were performed using the Origin Pro 2020 software (OriginLab).

## 3 Results

### 3.1 Collection of single-cell migration trajectories

**Figure 1:**
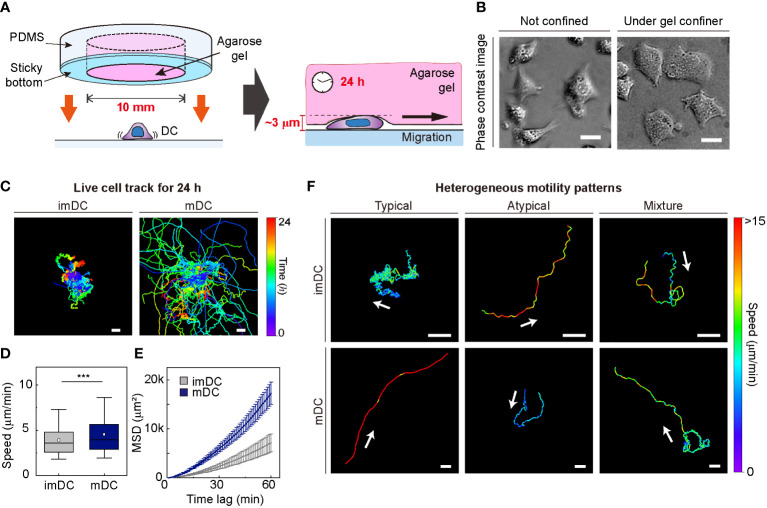
Heterogeneous dendritic cell (DC) motility under confinement. **(A)** Scheme of a gel confiner for an under-agarose assay with stable and reproducible confinement. The agarose gel (Averaged Young’s elastic modulus: 11.1 kPa) provided a tissue-like environment, and the sticky bottom beneath the PDMS structure enabled stable and reproducible confinement over a large area (78.5 mm^2^) **(B)** Phase-contrast images of DCs with and without the gel confiner. Scale bar = 10 μm. **(C)** 2D cellular migration trajectories of immature DCs (imDCs) and mature DCs (mDCs) for 24 h. The starting point of each trajectory was translated to the origin of the plot. Color codes indicate the track duration. Scale bar = 100 μm. One representative experiment out of three is shown. **(D)** Mean track speed of imDCs and mDCs. In the box plots, the bars include 95% of the points, the center corresponds to the median, and the box contains 75% of the data. Data were pooled from three independent experiments (imDC n = 460; mDC n = 371). The Mann-Whitney test was used to compare two groups. ***: *P*< 0.001. **(E)** Mean square displacement (MSD) curves of imDCs and mDCs. Lines indicate averaged MSD from three independent experiments, and the error bar indicates the S.E. **(F)** Examples of heterogeneous motility patterns of DCs. Motility directions are marked with arrows. The color codes indicate instantaneous speed. Scale bar = 100 μm.

To investigate the motile behaviors of DCs, we used primary mouse bone marrow-derived DCs (BMDCs) and characterized the DC phenotype (Materials and Method, **Supplementary Figure S2**). We used a gel confiner for long-term (24 h) monitoring of an unbiased population of freely migrating cells (**Figures 1A, B**). The gel confiner allowed for precise control over the degree of confinement in experiments involving multiple chips (**Supplementary Figure S3**). The detailed fabrication steps are described in our previous reports (13, 27). Cellular movements were imaged every 1 min using bright-field microscopy with a millimeter-scale broad field of view (1.3 × 1.3 mm^2^; **Supplementary Movies 1**, **2**). Using live-cell tracking data (**Figure 1C**), we found that mDCs showed faster and more persistent motility than imDCs, which is consistent with the results of previous studies (**Figures 1D, E**) (12, 13). However, outliers were consistently present (**Figure 1F**), and these atypical phenomena were often neglected in the pooled population analysis (**Supplementary Figure S1**).

Our ML kernel was designed to analyze individual, unperturbed, heterogeneous cellular motility. To construct the ML kernel, we prepared two independent datasets: one for training and the other for testing. The input data were pre-processed to remove trivial errors (Materials and Method). After pre-processing, we obtained trajectories of 301 (imDCs) and 348 (mDCs) for training and 460 (imDCs) and 371 (mDCs) for testing.

### 3.2 Developing the ML kernel to analyze DCs migration

**Figure 2:**
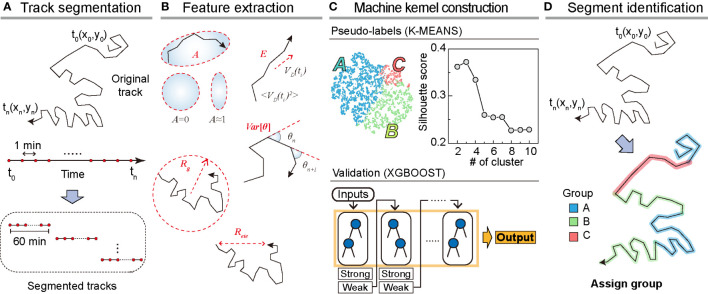
Overview of the machine learning method. **(A)** Track segmentation. We obtained 649 raw trajectories from the training datasets. After performing the data pre-processing, we segmented the trajectory into 1 h long pieces indexed with their start and end times. Using 5741 segmented trajectories, we made a trajectory pool. **(B)** Feature extraction. Using the segmented tracks, we calculated five features: radius of gyration ( $R_{g})$ , asphericity ( A) , energy consumption ( E) , end-to-end distance ( $R_{ete})$ , and variance of turning angles ( Var[θ]) . **(C)** The construction of the machine kernel. Using the input data, we performed K-means (32, 33) clustering to classify the trajectories using an unsupervised learning method and specify the label. Next, with the randomly chosen 2000 labeled trajectories, we trained XGBOOST (34) using supervised learning. The trained XGBOOST showed 99.6% agreement with the K-means clustering results for the label prediction. The hyperparameters were optimized using a cross-validated grid search in both methods. **(D)** Segment identification. Using the trained XGBOOST, we analyzed the original trajectory with a 1 h time window to identify the dynamic state of each segment.

To analyze dynamic heterogeneity in a single DC migration, we segmented the trajectory data into 1 h long tracks and obtained a total of 8787 tracks from the testing dataset (**Figure 2A**). For the training dataset, we prepared 5741 segmented tracks. From the segmented tracks, we extracted five features quantifying motility (radius of gyration, end-to-end distance, and average kinetic energy) and directionality (asphericity A and turning angle fluctuation Δθ ) as the input bases for the machine kernel (**Figure 2B**). The mathematical details of these features that enable quantification of the motility propensity and directionality of DC migration are as follows:

#### 3.2.1 Radius of gyration

To measure the second moments of positional fluctuations, we calculated the gyration tensor ${\mathbb{R}}_{ij}=\frac{1}{N^{2}}\sum_{t_{l}=1}^{N}\sum_{t_{m}=1}^{N}(r_{i}\left(t_{l}\right)-<r_{i}>)(r_{j}\left(t_{m}\right)-<r_{j}>)$ . Here, the symbol < O > denotes the mean, and the subscripts i,j represent the position coordinates x,y , respectively, and l,m denote the time index running from one to *N* where N is the trajectory length ( 50≤N≤60 ). The radius of gyration is obtained from the gyration tensor as follows:


(1) $$R_{g}\equiv \sqrt{Tr\mathbb{R}}.$$

Because the < $r_{i}$ > is the center of mass coordinate, *R_g_* represents the average spreading along the coordinates x and y .

#### 3.2.2 Asphericity

From ${\mathbb{R}}_{ij}$ we can calculate the spreading of the migration trajectory along its principal axis. This shape-related property, called asphericity, can be defined as


(2) $$A\equiv \frac{{\left(\lambda_{1}-\lambda_{2}\right)}^{2}\;}{{\left(\lambda_{1}+\lambda_{2}\right)}^{2}}$$

where the $\lambda_{1,2}\;$ is the eigenvalue of the gyration tensor ${\mathbb{R}}_{ij}$ . The asphericity is normalized in [0,1] such that the two limiting values represent a perfect circular shape for zero and a straight-line shape for unity.

#### 3.2.3 Energy consumption

The degree of spreading in equation (1) can be related to the total kinetic energy spent over the entire migration event. To measure the relationship between the spreading and energy consumption, we evaluated the average kinetic energy as follows:


(3) $$E\equiv \frac{1}{N-1}\sum_{n=1}^{N-1}V_{D}{\left(t_{n}\right)}^{2}.\;$$

Here, $V_{D}\left(t_{n}\right)=\frac{\sqrt{{\left(r_{x}\left(t_{n+1}\right)-r_{x}\left(t_{n}\right)\right)}^{2}+{\left(r_{y}\left(t_{n+1}\right)-r_{y}\left(t_{n}\right)\right)}^{2}}}{\left(t_{n+1}-t_{n}\right)}$ is the average migration speed at $t=t_{n}$ over 1 min.

#### 3.2.4 End-to-end distance

In addition to fast-spreading and high energy consumption, the directional propensity of migration can also affect the degree of overall spreading. We measured this effect in terms of the end-to-end distance:


(4) $$R_{ete}=\sqrt{{\left(r_{x}\left(t_{N}\right)-r_{x}\left(t_{0}\right)\right)}^{2}+{\left(r_{y}\left(t_{N}\right)-r_{y}\left(t_{0}\right)\right)}^{2}}$$

#### 3.2.5 Variance of turning angles

To measure the directional persistence, we have added a feature indicating the variance of turning angles. The θ is the angle between $\vec{D}\left(t_{n+1}\right)$ and $\vec{D}\left(t_{n}\right)\;$ in the range [−π,π) (**Figure 2B**), with a positive value in the counterclockwise direction where $\vec{D}\left(t_{n};\text{Δ}t\right)=\vec{r}\left(t_{n}+\text{Δ}t\right)-\vec{r}\left(t_{n}\right)$ . Using the segmented tracks, we calculated the variance of the turning angles as follows:


(5) $$Var\left[\theta \right]\equiv \frac{1}{N-2}\sum_{i=1}^{N-2}{\left(\theta_{i}-<\theta >\right)}^{2}.$$

### 3.3 ML-based discovery of three distinct modes of DCs migration

With the five extracted features from the segmented trajectories, we constructed the input data of size 5741×5 for training the machine (**Figures 2A, B**). First, we applied K-means unsupervised clustering (32, 33) to the input data to examine the number of distinct motility patterns of DCs. Next, we used the results from K-means clustering as pseudo-labels and cross-validated these using the supervised learning algorithm XGBOOST (34) (Materials and Method, **Figure 2C**). We point out that the combination of the unsupervised and supervised learning is advantageous when the cell-to-cell migration variation originates from temporally heterogeneous motion of a cell. After trained with the input data, the XGBOOST machine enables us to analyze unsegmented raw trajectories and decipher the migration pattern in the 1 h interval time windows along the trajectory (**Figure 2D**). We validated the ML method and its output by demonstrating that pattern classification is robust to variations in feature selection and their possible combinations.

In **Figure 2C**, we examined the average silhouette score to determine the number of distinct clusters in the migration patterns (31). The plot shows that the silhouette score reached the maximum when the number of distinct clusters was three if we used all five features introduced in our ML analysis. To assess the robustness of the results, we performed ML analysis with various combinations of input features. For this test, we used a training dataset of 5741 segmented trajectories. In **Supplementary Figure S4**, we evaluated the silhouette scores for five distinct combinations of the input features. For all cases, we confirmed that the DC migration data most likely consisted of three dynamic modes. We temporarily refer to the three groups as I, II, and III based on their population size.

**Figure 3:**
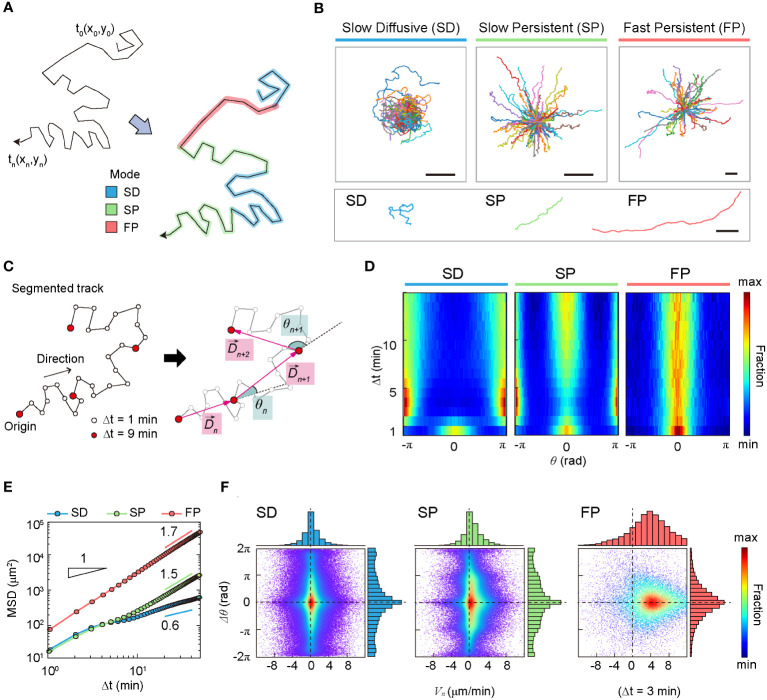
Statistical characteristics of three distinct machine-defined modes of dendritic cell (DC) motility. **(A)** Scheme of three distinct modes in a trajectory. **(B)** Sample trajectories of slow-diffusive (SD), slow-persistent (SP), and fast-persistent (FP) migration modes. Representative trajectories of each migration mode are shown together at the bottom panel (scale bar: 100 µm). **(C)** Schematic trajectories with the definition of the displacement vector $\vec{D}\left(t_{n};\text{Δ}t\right)$ and turning angle. The turning angle ranges from −*π* (clockwise turning) to  π (counterclockwise turning). In the phase-space density map, we measured the magnitude difference for successive displacement vectors $V_{n}=\left(\left|\vec{D}\left(t_{n+1};\text{Δ}t\right)\left|-\right|\vec{D}\left(t_{n};\text{Δ}t\right)\right|\right)/\text{Δ}t$ and their turning angle difference $\text{Δ}\theta_{n}=\theta_{n+1}-\theta_{n}$ . **(D)** The turning angle heat map (examples of individual trajectories are shown in **Supplementary Figure S8**). **(E)** The MSD curves for SD (blue), SP (green), and FP (red) modes. The guidelines show the anomalous exponent at long times. **(F)** The density map of phase space ( $V_{n},\text{Δ}\theta_{n}$ ) at Δt=3 min (examples at different time lags are shown in **Supplementary Figure S9**). The dots indicate data from a single-track segment. Dashed lines serve as visual guides. Data were pooled from three independent experiments for imDC and mDC.

The XGBOOST machine also allowed us to evaluate the statistical properties of the migration trajectories in each group with feature importance and the distribution of the five features (**Supplementary Figure S5**). It turns out that asphericity, *A*, is the most important feature among the five when determining the group assignment (**Supplementary Figure S5A**). It plays a critical role in differentiating group I from groups II and III (**Supplementary Figure S5B**). The features, $R_{g},\;\;R_{ete}$ , and E are effective in distinguishing group II and group III (**Supplementary Figures S5C–E**). These features quantify DC migration motility. In terms of $R_{g},\;\;R_{ete}$ , and E, groups I and II exhibited almost indistinguishable “slow” motility patterns, whereas group III, with high motility, differed from the other two groups. The variance of the turning angles showed a minor contribution, with a similar unimodal profile for all three groups (**Supplementary Figure S5F**). In **Table 1**, a few important dynamic features of groups I, II, and III are summarized. Based on averaged dynamic properties, we assigned the dynamic modes of groups I, II, and III to slow-diffusive (SD), slow-persist (SP), and fast-persist (FP) phenotypes, respectively (**Table 1**; **Figure 3** and **Supplementary Movies 3**-**5**). Note that the name has been given by the clustering results in five-dimensional feature space reflecting averaged dynamical properties, thus, slow and fast do not assign from their absolute velocity.

**Table 1:** Summary of the characteristics of each group (dynamic mode).

	Group I	Group II	Group III
Average speed ± Standard Deviation (μm/min)	2.58 ± 1.90	2.21 ± 1.61	6.27 ± 2.66
Anomalous exponent	0.59	1.47	1.67
Directionality	X	O	O
Dynamic mode	Slow-diffusive (SD)	Slow-persist (SP)	Fast-persist (FP)

DCs in the SD mode perform anti-persistent walks at a slow average speed, whereas FP mode cells show directional random walks at a fast average speed. The SP mode differed from the other two modes. It had a directional walk analogous to that of FP mode cells. However, the feature properties differed significantly from each other (**Supplementary Figure S5**). In terms of average speed, the SP mode was similar to the SD mode.

### 3.4 Migration dynamics of SD, SP, and FP modes

We investigated the dynamic properties of the SD, SP, and FP modes based on commonly used statistical observables, including mean-squared displacements (MSDs) and Van-Hove self-correlation functions. We also examined observables such as the turning angle heat map and density map of the phase space, $\left(V_{n},\text{Δ}\theta_{n}\right)$ . Additionally, we investigated the zigzag-like patterns of DC migration in detail.

#### 3.4.1 Mean-squared displacements

From a single trajectory, we calculated the MSD as a function of lag time Δt using the following definition:


(6) $$MSD=\frac{1}{T-\text{Δ}t}\int_{0}^{T-\text{Δ}t}{\left[\vec{r}\left(t+\text{Δ}t\right)-\vec{r}\left(t\right)\right]}^{2}dt\propto \text{Δ}t^{\alpha }\text{. }$$

Here the power-law exponent α is called the anomalous exponent and provides dynamic information on the diffusion process: 1) α=1 : the diffusion is normal. 2) 0<α<1 : diffusion is sub-diffusive. 3) 1<α<2 : Diffusion is super-diffusive. 4) α=2: The diffusion is ballistic such that the cell (or particle) moves at a constant velocity.

In terms of anomalous exponent, DCs in the SD mode are sub-diffusive, and DCs in the SP and FP modes are super-diffusive. The MSD plot indicates that cell migration dynamics change over time (**Figure 3E**). The measured values of α in the short- and long-time regimes differed for all three dynamic modes.

Compared with the averaged MSD (**Figure 3E**), the individual MSD curves showed a broad scatter for the SD and SP modes (**Supplementary Figure S6A**), indicating the prevalence of substantial cell-to-cell variation in the SD and SP migrations. We quantified the cell-to-cell dynamic heterogeneity in terms of P(α) (**Supplementary Figure S6B**). Notably, the corresponding distributions for the SD and SP modes showed wide spread from its peak value. The result indicates that most of the individual SD-mode DCs moved more persistently than expected based on the averaged quantity ( α≈0.6 ). For the SP-mode DCs, a few individual DCs exhibited sub-diffusive motion, even though the averaged MSD indicates super-diffusive movement with α≈1.5 .

FP mode migration was relatively homogeneous compared to the SD and SP modes. The P(α) was narrowly distributed around the peak value. A notable feature of the FP mode is that a few MSD curves show oscillatory behavior. We determined that such patterns could occur if the trajectory has a closed ellipse form in two-dimensional space. Although masked in the MSD plot, similar oscillating MSDs were found in the SD and SP modes as well.

#### 3.4.2 Distribution of displacements

The displacement probability density function (PDF) P(x|Δt) measures the probability of finding DC’s displacement in the interval [x, x+dx] over a given lag time where x=x(t+Δt)−x(t) is the ( x -component) displacement. As shown in **Supplementary Figures S7A**, **B**, we plotted the P(x|Δt) at several lag times for the three modes. For all migration modes, the PDFs were neither Gaussian nor followed a power law. The non-Gaussianity indicates that the SD and SP modes cannot be governed by Gaussian-based anomalous diffusion models, such as fractional Brownian motion and scaled Brownian motion (35). The exponential-like tail and cusp at the center in the PDFs strongly suggests that DC migration is temporally heterogeneous and shows cell-to-cell heterogeneity. The fluctuation in the instantaneous diffusivity of DC movement and its wide distribution may result in the cusp and the exponential-like tail, as reported in studies using the fluctuating diffusivity model (36, 37).

#### 3.4.3 Turning angle heat map

The turning angle θ(Δt) refers to the angle between two consecutive displacement vectors (**Figure 3C**). The displacement vector over a given lag time Δt is defined as $\vec{D}\left(t_{n};\text{Δ}t\right)=\vec{r}\left(t_{n}+\text{Δ}t\right)-\vec{r}\left(t_{n}\right)$ and $t_{n}=n\text{Δ}t$ . The turning angle was calculated as follows:


(7) $$\theta_{n}\left(\text{Δ}t\right)=\cos^{-1}\left(\frac{\vec{D}\left(t_{n+1};\text{Δ}t\right)\cdot \vec{D}\left(t_{n};\text{Δ}t\right)}{\left|\vec{D}\left(t_{n+1};\text{Δ}t\right)\right|\left|\vec{D}\left(t_{n};\text{Δ}t\right)\right|}\right)\;$$

where the θ∈[−π,π] , and the sign is positive (or negative) in the counterclockwise (or clockwise) direction, respectively. Next, we plotted the heat map of the normalized distribution for turning angles as a function of lag time. The corresponding heat maps for the three migration modes are shown in **Figure 3D**.

The heat map illustrates the change in directional persistence with time lag Δt (**Figures 3C, D**). Up to Δt ~ 3 min, the turning angle < θ > showed a peak at 0 , indicating that the cells keep moving in the same direction. This was true for all three modes. In contrast, for Δt>3 min, the SD migration mode exhibited a strong anti-persistent memory with two peaks at θ≈±π , which extended over several tens of minutes, resulting in sub-diffusive migration with an anomalous exponent α=0.6 (**Figure 3E** and **Supplementary Figure S6**). The SP migration mode was clearly distinguished from the SD. The strong peak at θ≈0 indicates that the SP mode is a directional walk consistent with its superdiffusive motion ( α=1.5 ) observed from the averaged MSD plot. Concurrently, the SP mode has the feature of anti-persistent walks ( θ≈±π ) (**Figure 1D**). We found that this seemingly contradictory migration pattern is a signature of zigzag directional walk; see our further analysis below. The FP mode migration mode showed a strong directional walk (**Figure 3B**). The heat map suggests that migration directionality is maintained for the total length of the trajectory (60 min) without significant dispersion of the angle fluctuation $<\theta^{2}>$ with increasing time (**Figure 3D**).

Additionally, we examined the heat maps obtained from a single trajectory (**Supplementary Figure S8**). For each dynamic mode, we plotted ten randomly chosen heat maps, which confirms that the pattern in the averaged heat maps (**Figure 3D**) is indeed observed at the single-trajectory level.

#### 3.4.4 Density map of phase space

To examine the radial and angular motions together, we constructed the phase-space $\left(\text{Δ}\theta_{n},\;V_{n}\right)$ where $\text{Δ}\theta_{n}=\theta_{n+1}-\theta_{n}$ , and $V_{n}=\frac{\left(\left|\vec{D}\left(t_{n+1};\text{Δ}t\right)\right|-\left|\vec{D}\left(t_{n};\text{Δ}t\right)\right|\right)}{\text{Δ}t}$ . **Supplementary Figure S9** shows scatter plots of $\left(\text{Δ}\theta_{n},V_{n}\right)$ for several lag times in the three modes. For the radial contribution, the SD mode was symmetric, whereas the other two modes were asymmetric. For the latter modes, $V_{n}$ is thicker on the positive side or shifted to the right, which indicates that the cell moved away from the origin with time.

For the angular part, the SP mode showed a thick shoulder ( ±π ) and small peaks at θ=± 2π for Δt≥3  min. When $\text{Δ}\theta_{n}\;$ has peaks at ±2π with a nonzero radial velocity, the corresponding trajectory shows two opposite turning events sequentially. The shoulder structure at ±π with a nonzero radial velocity may also indicate a zigzag-like motion, which will be described in the next section. The FP mode is characterized by a unimodal peak near zero, which shows a curved or straight motion by maintaining the turning angle for the overall relaxation time scale.

#### 3.4.5 Analysis of zigzag pattens in the DC migration data

A recent study proposed a zig-zag Lévy walk as a phenomenological motility model for DC migration (15). The zigzag Lévy walk is characterized by repeating persistent runs, where each run is composed of zigzag-like (i.e., left-right or right-left) random walks. Here, we systematically analyzed the DC trajectories to examine whether zigzag migration patterns were prevalent during the DC migration dynamics. In a nutshell, our analysis showed that the SP mode is a plausible candidate for defining empirically observed zigzag migration.

For the analysis, we constructed the phase space of successive turning angles $\left(\theta_{n},\;\theta_{n+1}\right),$ where the subscript n runs from the start-time segment to the last one. We showed the density maps of $\left(\theta_{n},\;\theta_{n+1}\right)$ at several lag times for the three dynamic modes (**Supplementary Figure S10**). The schematic in **Supplementary Figure S10B** shows the trajectory motifs corresponding to the nine specific dense spots in the density maps. From the density maps, we calculated a zigzag fraction (**Supplementary Figure S11**), which is the ratio of the total number of scatters between the first and third quadrants of the density map as a function of lag time. Note that our zigzag fraction is distinguished from the zigzag preference factor introduced in reference (15), in that our turning angles were defined from the displacement vectors of a given lag time, whereas, in reference (15), the turning events were determined using specific criteria, and the turning angles were obtained from these specific turning events.

The density maps in **Supplementary Figure S10C** show a few distinct patterns depending on the lag time and migration mode. At the shortest lag time ( Δt=1 min), a common feature for all three modes is that the phase densities are concentrated around the origin. This suggests that the directional persistence of migration movement is conserved in this short-time regime (**Supplementary Figures S10A, B**). This tendency is consistent with the fact that directional persistence is lost at Δt=3 min (**Figure 3D**).

In the SD mode, as lag times increase, the dense concentration in the phase density at the origin becomes dispersed. At Δt≥3 min, the density map became dense at the four corners (±π,±π ), indicating that there exists an abundant pool of two motifs: ( π,π ) and (−π,−π ), representing a circular motif, and ( π,−π ) and (−π,π ), indicating the presence of zigzag-like motifs (**Supplementary Figure S10B**). However, the SD mode is not a zigzag migration in the long run because zigzag movements do not result in a persistent run. The zigzag fraction (the ratio of the total number of scatters between the first and third quadrants in the density map) in the SD mode migration was always less than unity (**Supplementary Figure S11**).

The SP mode shows nine dense spots for Δt≥1 min. In addition to the four corners ( ±π,±π ) and the origin, a dense area exists at ( ±π,0 ) and ( 0,± π ). This corresponds to the thick shoulder structure observed in **Supplementary Figure S9**, and the motifs exhibit a zigzag motion (**Supplementary Figure S10B**). Because this phase space was obtained from two successive events, it does not directly represent zigzag migration. Nevertheless, the coexistence of a persistent motif and zigzag motif suggests that the SP mode is a class of zigzag migrations. This view is also supported by the zigzag fraction pattern, which is larger than unity (**Supplementary Figure S11**).

For the FP mode, the phase density always includes a concentrated region at the origin, which can be expected due to its strong persistent movement over the entire observation period. The zigzag fraction increased to ~1.1, subsequently decreasing to saturation around unity (**Supplementary Figure S11**). It can be inferred that after a long relaxation time, the trajectory shape is curved due to strong directional persistence, indicating that the zigzag patterns are smeared (or show weak wiggling) owing to the large curves.

Based on the above analyses, we recapitulate the dynamic characteristics of the three modes in the following: The SD migration showed a non-Gaussian subdiffusion pattern (**Supplementary Figure S7**) and differs from a zigzag-type anti-persistent walk. The phase-space density map $P\left(\text{Δ}\theta_{n}\right)$ shows a peak at 0 and $P(V_{n})$ is symmetric at 0 (**Figure 3F** and **Supplementary Figures S8**, **S9**), indicating that successive displacements preserve the same turning angles. Accordingly, SD migration was curly, as displayed in the exemplified trajectories (**Figure 3B**).

The SP mode exhibited a zigzag-like persistent walking pattern (**Figure 3B**). Interestingly, in the turning angle heat map, DCs show features of both persistent ( θ≈0 ) and anti-persistent ( θ≈±π ) walks (**Figure 3D**). The MSD analysis shows that SP migration is super-diffusive with α≈1.5 for Δt>10 min (**Figure 3E**). For Δt<10 min, the SP mode of migration was similar to that in the SD mode. SP cell migration was highly heterogeneous (**Supplementary Figure S6**), and showed non-Gaussian diffusion with a displacement PDF similar to that of SD motion (**Supplementary Figure S7**). The phase-space maps and zigzag preference factor demonstrated that SP migration shows an empirically observed zigzag pattern (**Figure 3F** and **Supplementary Figures S9**-**11**).

The FP migration mode showed a strong directional walk (**Figure 3B**). Migration directionality is maintained for the total length of the trajectory (60 min) without significant dispersion of the angle fluctuation $<\theta^{2}>$ with increasing time (**Figure 3D**). FP migration is not ballistic but super-diffusive with α≈1.7 (**Figure 3E**), indicating faster and more directional motility than the SP mode. Unlike the SD and SP modes, FP migration dynamics are homogeneous (**Supplementary Figure S6**). Interestingly, P(Δx|t) is an exponential distribution, indicating that a power-law tail does not exist. Actively diffusing biological particles often exhibit a power-law PDF of displacement as a signature of Lévy walks. Examples include *Escherichia coli* (i.e., run-and-tumble micro-swimmers) (5), motor-driven macromolecules in the cytoplasm (38), mRNA-protein complexes (39), foraging birds (40), and chemokine-stimulated immune cells (CD8+ T cell) (6). However, the results of our analysis indicate that DC migration dynamics do not belong to the class of Lévy walks, regardless of maturation (**Supplementary Figure S7**).

### 3.5 Actin nucleation and contraction affect the migratory mode distribution of imDCs and mDCs

**Figure 4:**
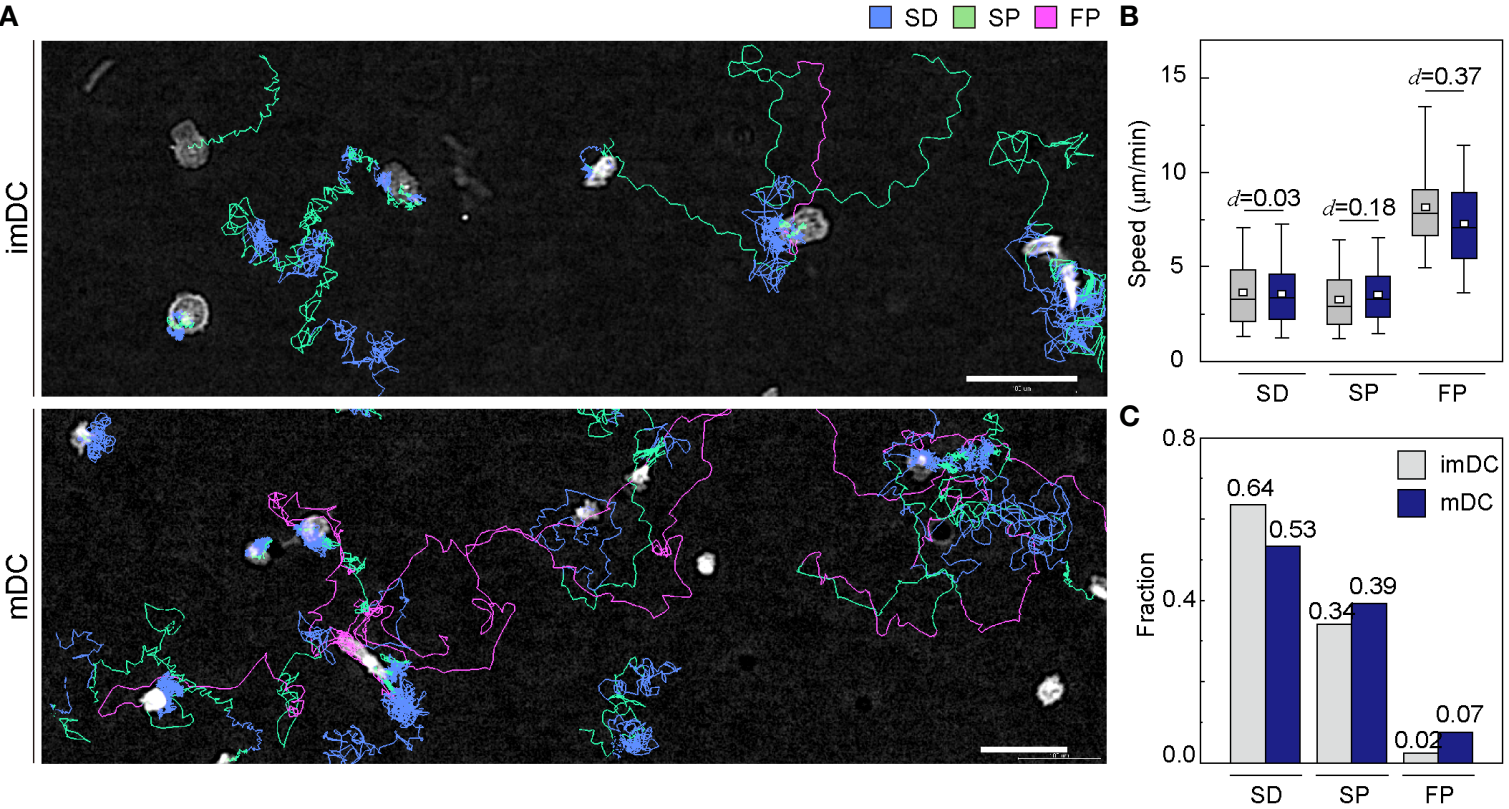
Distribution of migration modes. **(A)** Representative images depicting the mode assignment for the individual cell migration track. Each line represents the migration mode for the cellular migration trajectories, with colors indicating SD (blue), SP (green), and FP (red) modes. Background subtraction was performed to increase the contrast of the bright-field image (scale bar = 100 µm). **(B)** Mode mean track speed of immature DCs (imDCs) and mature DCs (mDCs). Data were pooled from three independent experiments. (SD: imDC n = 3660 ; mDC n = 1624, SP: imDC n = 1953 ; mDC n = 1192 , FP: imDC n = 132 ; mDC n = 226 ). Cohen’s *d* (*d*) was used to indicate the standardized difference between the two means. **(C)** Occurrence frequency of SD, SP, and FP modes in the imDC and mDC motility data.

To investigate how the maturation status of DCs influences the distribution of the three migration modes, we assigned the migration mode 1 h interval traces over time until the cell finally escaped from the field of view (**Figure 4A** and **Supplementary Movies 6**, **7**). The speed and fraction of the three migration modes suggest that DC migration occurs mostly in the SD and SP modes, and their speeds are unaffected, regardless of their maturation status (**Figures 4B, C**). The FP mode was more frequently observed in mDCs than in imDCs.

**Figure 5:**
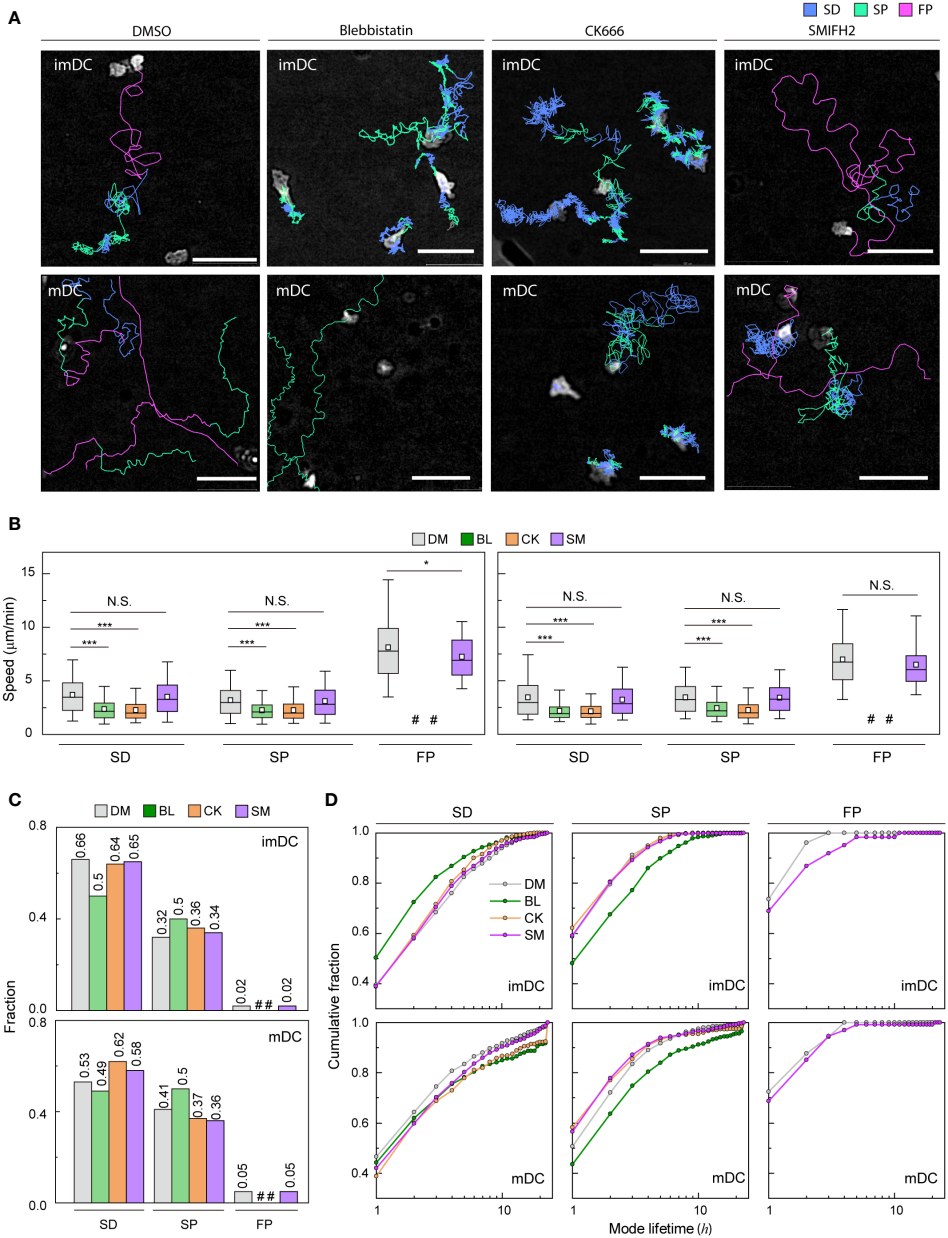
Molecular inhibition in motility mode dynamics **(A)** Representative images of imDCs and mDCs under the effect of drugs. Each line represents the migration mode for the cellular migration trajectories with colors indicating SD (blue), and SP (green) modes. Background-subtraction was performed to increase the contrast of the brightfield image (scale bar = 100 µm). **(B)** Mean track speed of imDCs and mDCs after treatment with DMSO ( 0.1 %), 20 μM blebbistatin (BL, myosin II inhibition), 100 μM CK666 (CK, Arp2/3 inhibition) and 12.5 μM SMIFH2 (SM, Formin inhibition). In the box plots, the bars include 95% of the points, the bar corresponds to the median, square corresponds to mean, and the box contains 75 % of the data. Data were pooled from three independent experiments (imDC: SD, DM n = 3702 , BL n = 1491 , CK n = 2910 , SM n = 3760; SP, DM n = 1815 , BL n = 1508 , CK n = 1613 , SM n = 1957; FP, DM n = 99 BL n = 11 , CK n = 15 , SM n = 102; mDC: SD, DM n = 1588 , BL n = 607 , CK n = 973 , SM n = 2371; SP, DM n = 1228 , BL n = 612 , CK n = 584 , SM n = 1474; FP, DM n = 162 , BL n = 10 , CK n = 9 , SM n= 217);. The Kruskal-Wallis test with Dunn’s *post hoc* analysis was used to determine statistical significance. *: *P* <0.05,  ***: *P* <0.001 , N.S.: *P* >0.05 , #: very low incidence. **(C)** Occurrence frequency of SD, SP, and FP modes for imDC and mDC migration after drug treatment. **(D)** Cumulative distribution of consecutive mode lifetime for imDCs and mDCs migration after drug treatment.

Cell speed and persistence are controlled by the regulation of actin dynamics (41, 42). Because myosin II-mediated contractility and actin polymerization key mechanism of cell motility (43), we sought to test how actin mediators influence migration mode distribution (**Figure 5A** and **Supplementary Movies 8**-**15**). During experiments, they survived similarly to the controls over the next 24 h (**Supplementary Figure S12A**).

Although the overall speed of both imDCs and mDCs was reduced (**Supplementary Figure S12B**), which is consistent with previous studies (14, 44–46), the FP mode was absent, and the mean speed of both SD and SP modes was decreased significantly for both cases of myosin II and Arp2/3 inhibition (**Figure 5B**). Remarkably, myosin II inhibition increased the SP mode of migration in the imDC population (**Figure 5C**), whereas Arp2/3 inhibition increased the SD mode of migration of mDCs. Therefore, after the inhibition, mode fractions seem to converge to a similar value irrespective of maturation status.

Next, we determined the mode lifetime, length of mode continuity, and how molecular inhibition influenced the mode longevity. Interestingly, myosin II inhibition reduced imDC SD mode durations while increasing mDC SD and SP mode lifetimes (**Figure 5D**). Arp2/3 complex inhibition enhanced the lifespan of mDCs in the SD mode but not that of imDCs, demonstrating that mode lifetime dynamics and the control of actin dynamics are highly dependent on DC maturation status.

Unlike myosin II and Arp2/3 inhibition cases, the FP mode was maintained in DCs treated with a Formin inhibitor, SMIFH2 (12.5 μM) (**Figure 5A**). While the speed of the SD and SP modes was not changed much, there was a decrease in the speed of the imDC FP mode, but not in mDC (**Figure 5B**). Analysis of the mode fraction showed that the ratio of SD and SP mode was comparable to Arp2/3 inhibition cases. However, FP mode was preserved only in DCs treated with SMIFH2 (**Figure 5C**). It is noteworthy that SMIFH2 treatment also impacts mode lifetime. A comparable pattern to CK666 treatment was observed when DCs were treated with SMIFH2. However, an increase in the FP mode lifetime of imDC was observed, whereas no such change was detected in mDC (**Figure 5D**).

### 3.6 Maturation status-specific migratory mode transition dynamics

**Figure 6:**
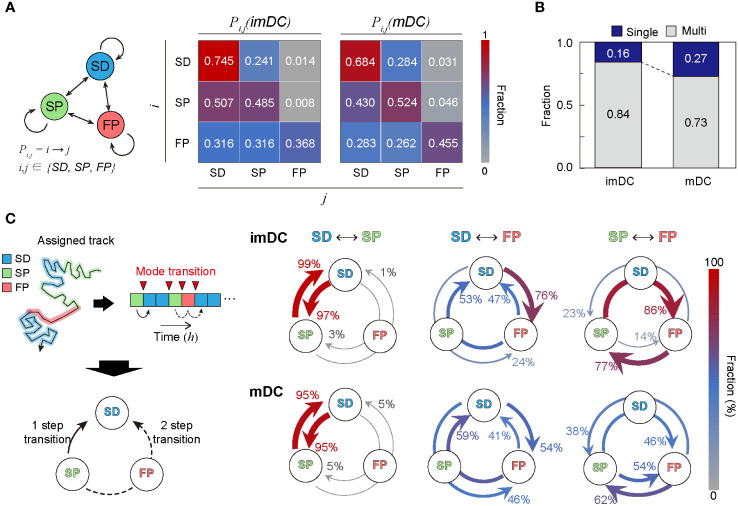
Transition dynamics of migration modes. **(A)** Transition model between the three migration modes. *P_ij_* denotes the transition rate from modes i to j (including self-transition) where i,j∈{SD,SP,FP} . **(B)** Fraction of homogeneously (non-transitionary) and heterogeneously (transitionary) migrating cells over the observed duration. **(C)** The time trace of migrating modes is depicted graphically, as well as the time points at which the mode shift occurs (red inverse triangle). The mode transition may occur in one step (e.g., SP→SD) and two steps (e.g., SP→FP→SD). The probabilities of a one-step or two-step mode transition from modes *i* to *j* ( i≠j ) were evaluated and plotted together for the cross-transition between different migration modes. The transition, including the FP mode, shows distinct pathways depending on the maturation level. imDCs showed the cyclic mode transition (SD→FP→SP→SD), whereas mDCs had a more bidirectional transition. Data were pooled from three independent experiments. All plots shown here were obtained from three independent experiments.

To study the transition dynamics of the DC migration modes, we constructed a transition matrix (**Figure 6A**) that showed several noteworthy features. First, self-transition predominates, signifying that DCs move with strong mode persistence. Compared with imDCs, mDCs have higher self-transition rates for the SP and FP migration modes and lower self-transition rates for the SD migration mode. This tendency explains why mDCs moved more persistently than imDCs; mDCs avoided SD migration in favor of SP or FP migration. Second, SD was the most recurrent migration mode for both imDCs and mDCs. The highest sum of influx rates was observed for the SD mode ( $\sum_{i\in \left\{\text{SD},\text{ SP},\text{ FP}\right\}}P_{i\to SD}$ ). Third, maturation increases the transition to FP mode. Comparing the FP column of the transition matrix of imDCs and mDCs, we found that every component of the transition from SD, SP, and FP to FP was significantly increased.

Irrespective of maturation, DC migration is dominated by two slow modes (SD and SP), suggesting that DCs usually move at a slow speed with or without directional persistence. The high energy-consuming FP migration mode was observed intermittently, and the frequency of occurrence increased after maturation. In **Figure 6B**, we plotted the population fraction of mode-preserving (single-mode) and mode-changing (multi-mode) cellular migration tracks. Interestingly, mDCs have a larger fraction of single-mode migration tracks than imDCs, indicating that maturation participates in stabilizing the innate migration of DCs.

Next, we examined the cross-transition dynamics among different migration modes (**Figure 6C**). Mode changes can occur in one or two steps. We emphasize that the cross-mode transition diagram should be distinguished from the total transition matrix, as shown in **Figure 6A**. For both imDCs and mDCs, a single-step change was the dominant pathway for the transition between the SD and SP modes. For the transition from the SD to FP modes, the one-step pathway was the most common for imDCs. For mDCs, the fractions for the one-step (SD→FP) and two-step (SD→SP→FP) transitions were similar. From the SP to FP transition, the two-step transition (SP→SD→FP) was the primary pathway for imDCs, whereas both one- and two-step transitions were similar for mDCs. Taken together, imDCs predominantly followed the unicyclic SD→FP→SP→SD transition (**Supplementary Movie 16**). In contrast, mDCs showed no transition directionality.

## 4 Discussion

Our results suggest that ML-assisted analysis can successfully identify the heterogeneous motility patterns of DCs. We found that the overall diffusive and persistent cellular motion of DCs are emergent properties of the dynamic switching of the three migratory modes, SD, SP, and FP, controlled by intracellular actin dynamics. Emerging literature on both experimental and modeling studies on innate DCs migration has reported dynamic switching between diffusive and persistent motility (9, 14, 45, 47, 48). For instance, the universal coupling between cell speed and cell persistence model could explain the general trend that faster cells tend to turn less, which successfully predicted the three typical patterns of experimental cell trajectories; diffusive, persistent, and intermittent (45). When imDCs were confined in a 2D chip or a 3D collagen gel, they exhibited two distinct migration states: persistent and diffusive migration (14, 45). A subsequent study reported that the persistence-speed coupling of DC motility enhances the search efficiency (48). In addition, a zigzag generalized Lévy walk model incorporating both the zigzag-turning walk and the intermittent walk characteristics was proposed to explain the search strategy of DCs (15). However, it should be noted that the time scales varied between studies. At very short time scales, cellular mobility could always be persistent, yet at very long-time scales, it might be diffusive. DCs migration trajectories were often collected at 3 min intervals for ~ 15 h (14, 45, 48), whereas the zigzag generalized Lévy walk model used 10 min-long trajectories collected at 10 sec time intervals (15). In addition, many distinct interpretations of directional persistence have been implemented (12, 14, 15, 45, 47, 48). For instance, if a 12-minute segment of a trajectory collected with 3 min time interval contained less than two abrupt directional changes, it was classified as persistent, where abrupt means the turning angle between two consecutive displacement vectors is greater than 90 degrees (14, 45). The path persistence, the length of the cell path divided by the diameter of the theoretical circle that holds the whole trajectory, is 1 for highly persistent trajectories and 0 for purely random migration tracks (12). In this study, we used an unsupervised ML technique to classify the cell motility patterns using one-hour-long cell migration tracks segmented from trajectory data collected for ~ 24 h at fine time intervals of 1 min, which allowed us to avoid using an arbitrary definition of diffusive or persistent motility.

Remarkably, our analysis suggests that imDCs changed their migration modes more frequently, and predominantly followed a unicyclic SD→FP→SP→SD transition, indicating that imDCs rapidly increase their speed during the shift from diffusive to persistent motility; however, persistence progressively declines when switching back to diffusive motility. In contrast, mDCs show no transition directionality. Based on these findings, we hypothesized that imDCs may evacuate the antigen-cleared location quickly and subsequently slow down gradually to find a new site to set before displaying diffusive motility to acquire antigens. Even though the time scale was much shorter (< 30 min), faster acceleration than deceleration of imDC motility was also noted in the 1D channel (47). Although persistence-speed coupled biphasic intermittent random walks have been shown to boost the search efficiency of imDCs (48), further analysis of the roles of the SP mode in the deceleration process is required.

DCs movement under confinement is commonly referred to as “amoeboid.” This is because the movement is independent of adhesion molecules but driven by protruding actin network at the front and squeezing contractions of actomyosin at the back of the cell (41). However, the molecular processes that govern the dynamic oscillation of intrinsic DC motility patterns in the absence of an external stimulus are not fully understood. Recent studies report that polarization due to spontaneous actin polymerization waves (14) and intracellular trafficking of myosin II (47) plays a key role in deterministic alteration between different migration modes of imDCs. Our findings that mDCs have more SP and FP mode motility than imDCs align with previous research, showing that DC maturation induces inherent changes in motility, resulting in faster and more persistent migration overall (12). When we examined the involvement of myosin II and Arp2/3 in three migratory modes of imDCs and mDCs, we discovered that myosin II or Arp2/3 inhibition entirely eliminated the FP mode and considerably lowered the mean speed of both the SD and SP modes. Myosin II inhibition increased the SP mode of migration in the imDCs, which is in agreement with previous report demonstrating reduced speed and increased persistence of imDC migration when Myosin II activity was interfered with Y27632 (14). Our results indicate that the increase in SP mode is more pronounced in imDCs because their original motility is more diffusive than that of mDCs. The SD mode of migration, on the other hand, was increased in mDCs by blocking the Arp2/3 complex. For imDCs, which are inherently more diffusive, the effect was minimal.

A previous report suggested that directional persistence is the cumulative effect of several interconnected feedback loops that influence actin polymerization at the leading edge (49). The Arp2/3 complex has been reported to play diverse roles in cellular motility, such as generating mechanical cues for cell polarization (49), facilitating nuclear deformation (50), and facilitating migration in dense tissues by forming mechanosensitive actin patches (46). Thiam et al. demonstrated that perinuclear Arp2/3-driven actin polymerization facilitates DC migration by deforming the nucleus, allowing the cell to squeeze through narrow gaps in the extracellular matrix (50). Additionally, Gaertner et al. showed that the Wiskott-Aldrich syndrome protein (WASp), which activates the Arp2/3 complex, triggers mechanosensitive actin patches that facilitate immune cell migration in dense tissues by sensing and responding to the mechanical properties of the environment (46). Hence, when DCs are subjected to a gel confiner that imitates physiological mechanical load and confinement, treatment with CK666 fails to elicit a response to physical influence, causing DCs to lose their capacity to survey their environment. Our findings support this idea, as they demonstrate that the inhibition of Arp2/3 reduces the persistence of movement in both SP and FP modes.

Formin regulates actin polymerization by generating linear actin filaments, unlike Arp2/3 which produces branched actin networks. Our findings align with a recent study (14) that showed Arp2/3 inhibition reduces persistent migration, while Formin inhibition leads to a comparable distribution of diffusive and persistent migration similar to control DCs. The study also highlighted the importance of both Arp2/3 complex and Formin proteins in regulating actin dynamics and cell polarization, and demonstrated their cooperative role in generating actin waves that drive cell migration (14). Inhibiting Arp2/3 or formins had a significant impact on actin polymerization waves, where Formin inhibition led to a higher nucleation rate and shorter-lived waves, while Arp2/3 inhibition entirely suppressed wave formation.

Future studies should extend the current ML-based classification strategy to investigate DCs migration under different biological, chemical, or physical stimuli, and to examine the effect of other DC subtypes. While GM-CSF derived BMDCs are commonly used *in vitro* models for studying DC motility (9–14), fms-related tyrosine kinase 3 ligand (Flt3L)-derived BMDCs have been proposed as a more *in vivo*-like model (51–53). As Flt3L and GM-CSF are essential regulators of DC development *in vivo*, it would be intriguing to study the differences in DC motility dynamics between these two cell types.

DC motility is also affected by antigens. When DCs encounter danger signals, such as pathogen-associated molecular patterns (PAMPs) that are recognized by Toll-like receptors (TLRs), they can respond by changing their motile behavior (54). For example, it has been shown that different TLR ligands elicit distinct patterns of DC migration, with some ligands inducing faster and more directional migration than others. Furthermore, the duration of antigen stimulation can also impact DC motility (13). Moreover, a recent study has demonstrated that extracellular ATP may accelerate DC mobility, suggesting that DCs may be more effective at antigen uptake through ATP released by infected or dead cells (55). In addition, it has also been shown that self-antigens may trigger such motility changes. Recent research has shown that the dynamics of DC macropinocytosis, which are regulated by Myosin II, are connected to cell migration dynamics and may improve searching efficiency (9). Using ovalbumin as an antigen, this study demonstrates that the antigen capture mechanism may govern DC motility dynamics even in the absence of PAMPs or damage-associated molecular patterns (DAMPs). Consequently, investigating DC motility dynamics in response to various antigens can provide new insights into the complex interplay between DCs and the immune system, and could have important implications for developing novel immunotherapies and vaccines.

Furthermore, microscopic features and molecular mechanism responsible for dynamic switching of different migration modes remains to be investigated. Recent studies have shown that the stability of actin intercellular flow is a crucial feature for maintaining their lifespan of motility on a microscopic scale, and a number of molecules, including myosin II, Arp2/3 and Formin, are referred to explain the process (14). In the present study, we noticed that blebbistatin administration altered the hour-scale mode lifetime in both imDCs and mDCs. For CK666, the mode lifetime shift was much larger in mDCs than in imDCs. In contrast, SMIFH2 uniquely shift FP mode lifetime in imDC. These results may indicate that the regulation of mode transition varies depending on the maturation status.

We acknowledge the limitation of this study. Firstly, it was necessary to pool the data from a minimum of three independent experiments to achieve sufficient sample numbers for ML analysis. Although the GM-CSF derived BMDCs used in each experiment may differ, we could not collect a sufficient number of cells without pooling because we used a small number of DCs per chip (~ 10 cells/mm^2^) to minimize the influence of neighboring cells because this study aims to investigate innate DC random motility in 2D space in the absence of external stimuli. In addition, the data from cells that inevitably interacted (5.36%) were excluded from the analysis. This approach may not reflect natural cell behavior in-vivo, but it may help understand the fundamental principles of cell migration and study the impacts of individual stimuli. Secondly, the number of FP mode tracks was much smaller than SD or SP mode because fast migrating cells easily escape from the field of view. Despite these constraints and the variation between and across datasets, we identified distinctive migration modes both from cross-validation of training data and independent testing data sets.

In summary, ML-enabled discovery of the history-dependent mode transition of motile cells provides a new paradigm for understanding complex cellular motility as an alternative to the current analysis of memoryless diffusive particles. We envision this approach can be further utilized to understand the complex dynamics of cellular migration in response to external stimuli, such as chemokines, physical confinement, or environmental stiffness, as well as pathogens and cancer cells.

## Data availability statement

The raw data supporting the conclusions of this article will be made available by the authors, without undue reservation.

## Ethics statement

The animal study was reviewed and approved by Institutional Animal Care and Use Committee of the Ulsan National Institute of Science and Technology (UNISTIACUC-19-15).

## Author contributions

All authors contributed to the article and approved the submitted version. YC performed the experiments and analyzed the data. TS performed the machine learning analysis with input from all authors. J-HJ and Y-KC supervised the project.
