# Supplementary material for: A machine learning approach to discover migration modes and transition dynamics of heterogeneous dendritic cells
Source: Front Immunol. 2023 Apr 4;14:1129600. doi: 10.3389/fimmu.2023.1129600 (PMC10110959; doi:10.3389/fimmu.2023.1129600)
Supplement: Supplementary file 1 [file DataSheet_1.pdf]

Supplementary Material

A machine learning approach to discover migration modes and transition dynamics of heterogeneous dendritic cells

Taegeun Song †, Yongjun Choi †, Jae-Hyung Jeon*, and Yoon-Kyoung Cho*

† These authors contributed equally to this work.
* **Correspondence:** Jae-Hyung Jeon and Yoon-Kyoung Cho
Email: jeonjh@gmail.com (J-H. J.); yoonkyoung.cho@gmail.com (Y-K. C.)

Supplementary Figures

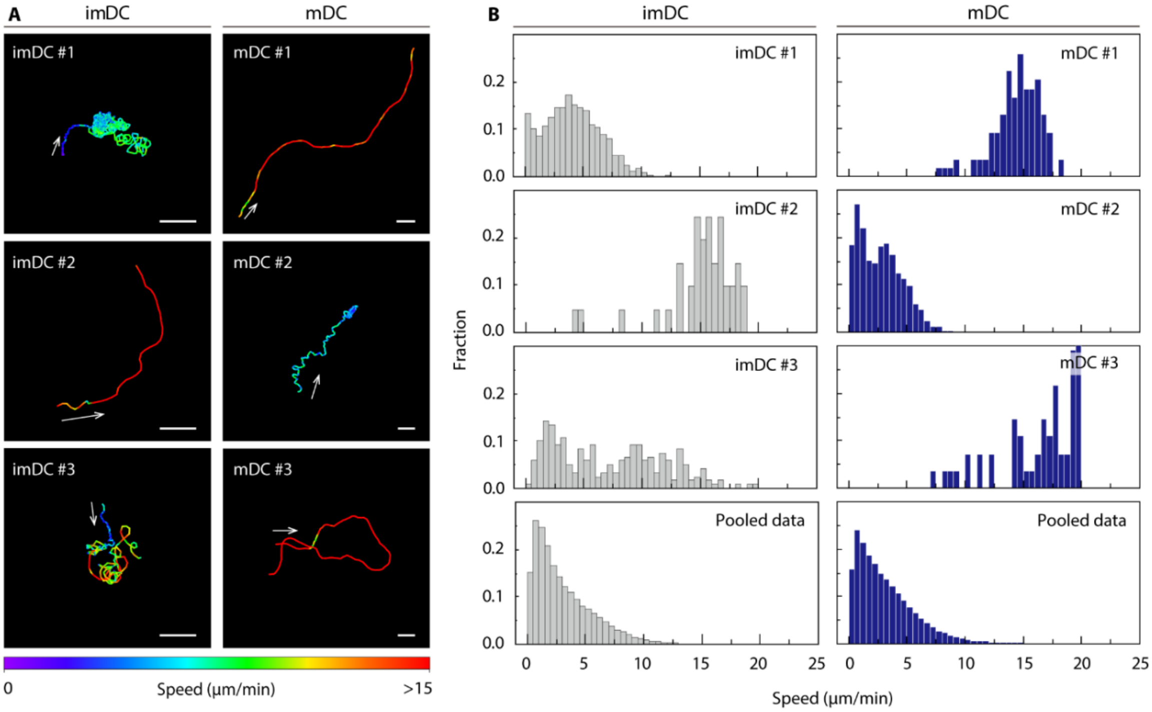

Supplementary Figure 1.

Dendritic cell (DC) heterogeneous tracks and missing minor tracks in the averaged result. Even in the same maturation status, atypical motilities are identified in the population. **(A)** Additional representative tracks are color-coded. The color indicates the instantaneous speed. **(B)** Distribution of instantaneous speed from **(A)**. Single-cell scale tracks showed heterogeneous motility patterns; however, this heterogeneity was absent in averaged results from whole cells (pooled data). Single-cell tracks were collected from one of three experiments (imDCs: n = 93, mDCs: n = 94).

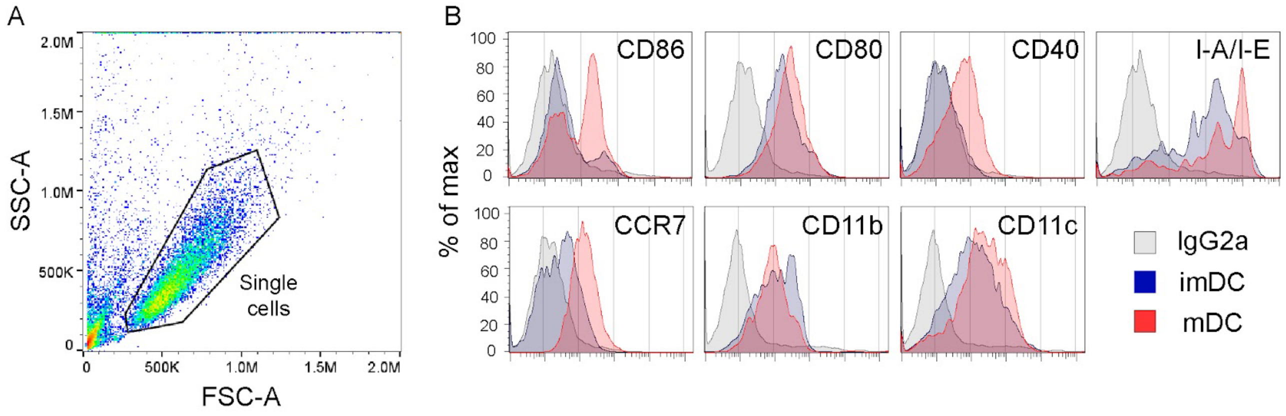

Supplementary Figure 2.

DC characterization. Flow cytometry analysis was performed for all independent experiments to confirm the DC phenotype. **(A)** Gating strategy for Flow cytometry analysis. Cell debris and doublet were excluded from the population. This gating strategy was applied to both imDC and mDC. **(B)** Co-stimulatory molecules (CD86, CD80, and CD40), antigen-presenting molecules (MHC class II (I-A/I-E)), chemotaxis receptor (CCR7), and DC markers (CD11b and CD11c) were evaluated in both imDCs and mDCs. One representative experiment out of three is shown.

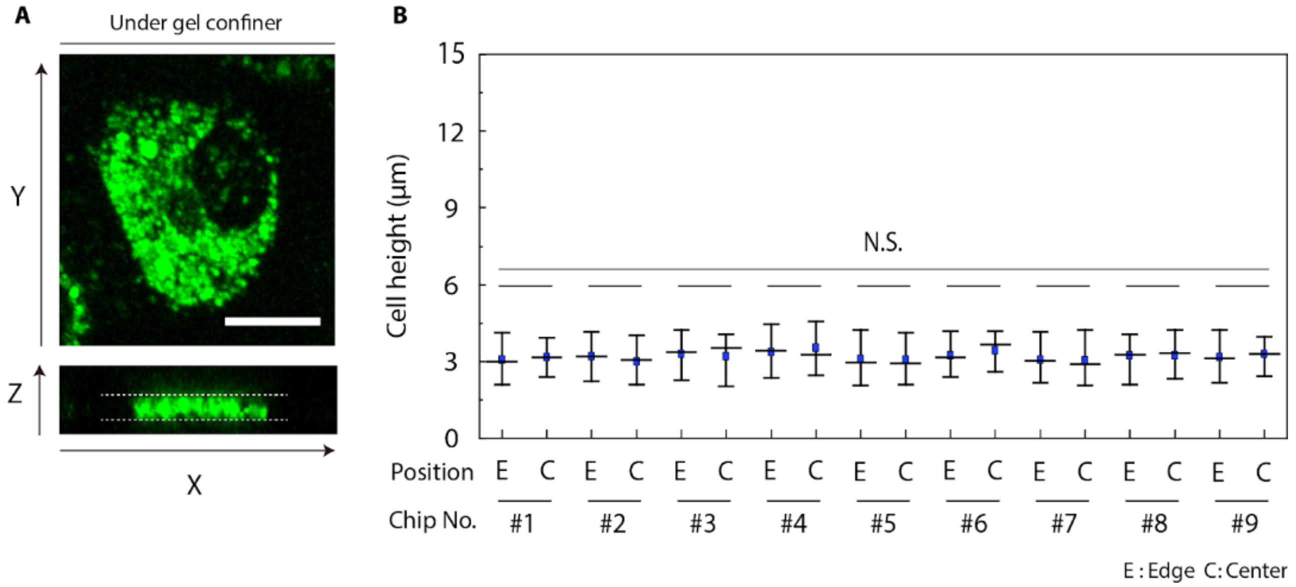

Supplementary Figure 3.
Uniform degree of confinement regardless of the cell position under gel confiner. (A) 3D confocal microscopic image of DiO stained DCs (Scale bar = 10 μm). Cell height was measured to check the degree of confinement. **(B)** The reproducibility of the gel confiner chip (r = 5 mm) was evaluated by measuring cell height in nine different chips. Each chip had five measurement positions, including one at the center (r = 0 mm) and four at the edges (r = 4 mm). More than 20 cells were measured at each position. In the plots, bars include 95% of the data, the central bars indicate the medians, and blue dots indicate the means. Mann-Whitney statistical test was applied to compare the populations between the center and edge positions. Kruskal–Wallis/Dunn's multiple comparisons testing was used to compare different sample populations; N.S.: P > 0.05.

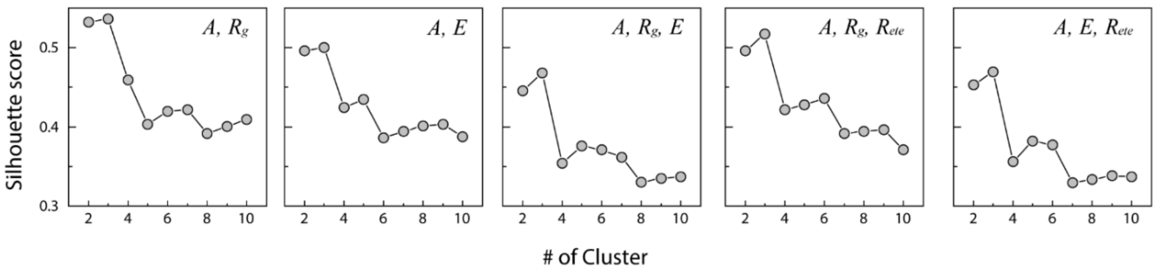

Supplementary Figure 4.

The number of distinct groups obtained using an averaged silhouette score. The number of groups is shown as a function of the number of clusters using K-means clustering with five distinct combinations of the original features. The feature combination is annotated in the legend. In the plots, all features were normalized using the MinMaxScaler in the range of [0,1] provided by the scikit-learn package in Python(1)

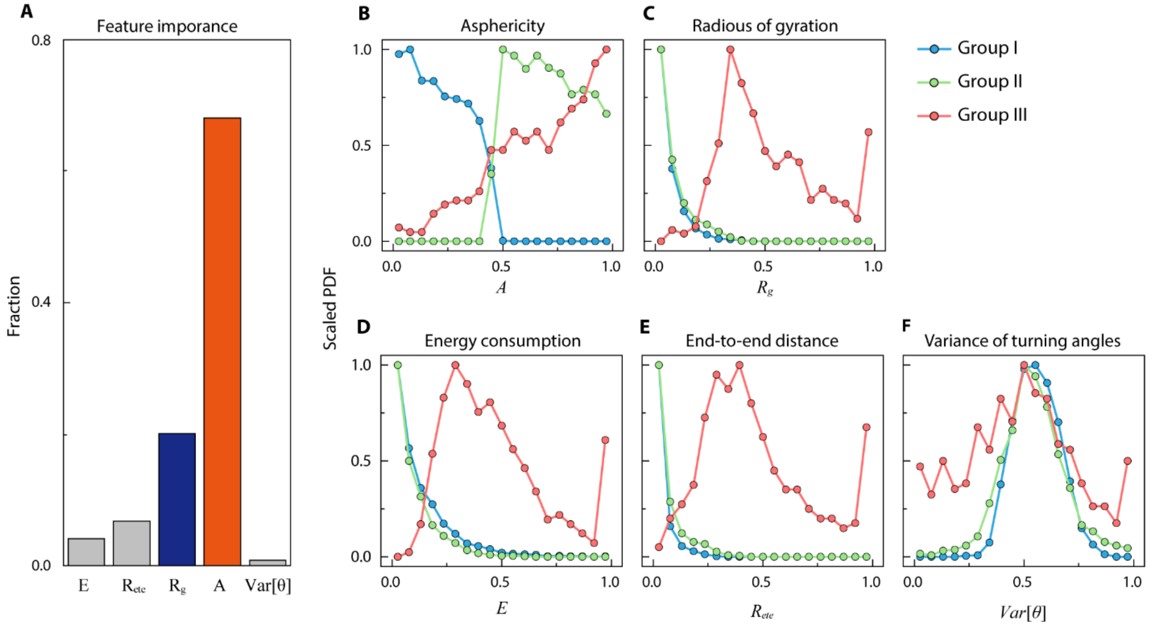

Supplementary Figure 5.

Feature importance and feature distribution for three groups. (A) The feature importance (%) relative to the five features used in machine learning kernel training. The trained algorithm extracts the distributions of five features for each group, including Asphericity (**B**), Radius of gyration (**C**), End-to-end distance (**D**), Energy consumption (**E**), and Variance of turning angles (**F**). The color indices are blue (group I), green (group II), and red (group III).

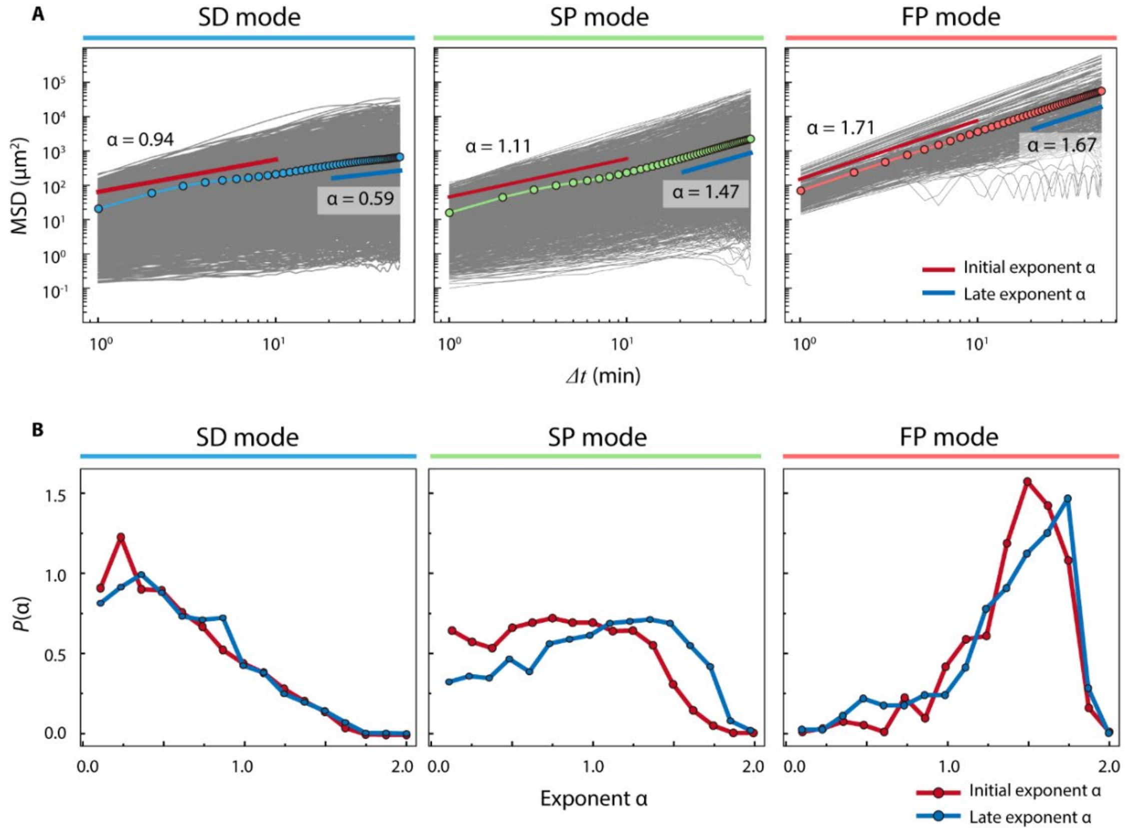

Supplementary Figure 6.

Mean-squared displacement (MSD) curves and the distribution of anomalous exponent α for the slow-diffusive (SD), slow-persistent (SP), and fast-persistent (FP) modes. **(A)** In the MSD plot, the gray lines depict the individual MSD curves, and the symbol with the solid line indicates their average. We obtained two fitted anomalous exponents from the averaged MSD: The short-time exponent for $\Delta t \in [0,10]$ min (initial) and the long-time exponent for $\Delta t \in [20,50]$ min (late). **(B)** The distribution of the fitted anomalous exponents $\boldsymbol{\alpha}$ from the individual MSD curves in (A). Each panel shows two $P(\alpha)$ for the short- and long-time regimes.

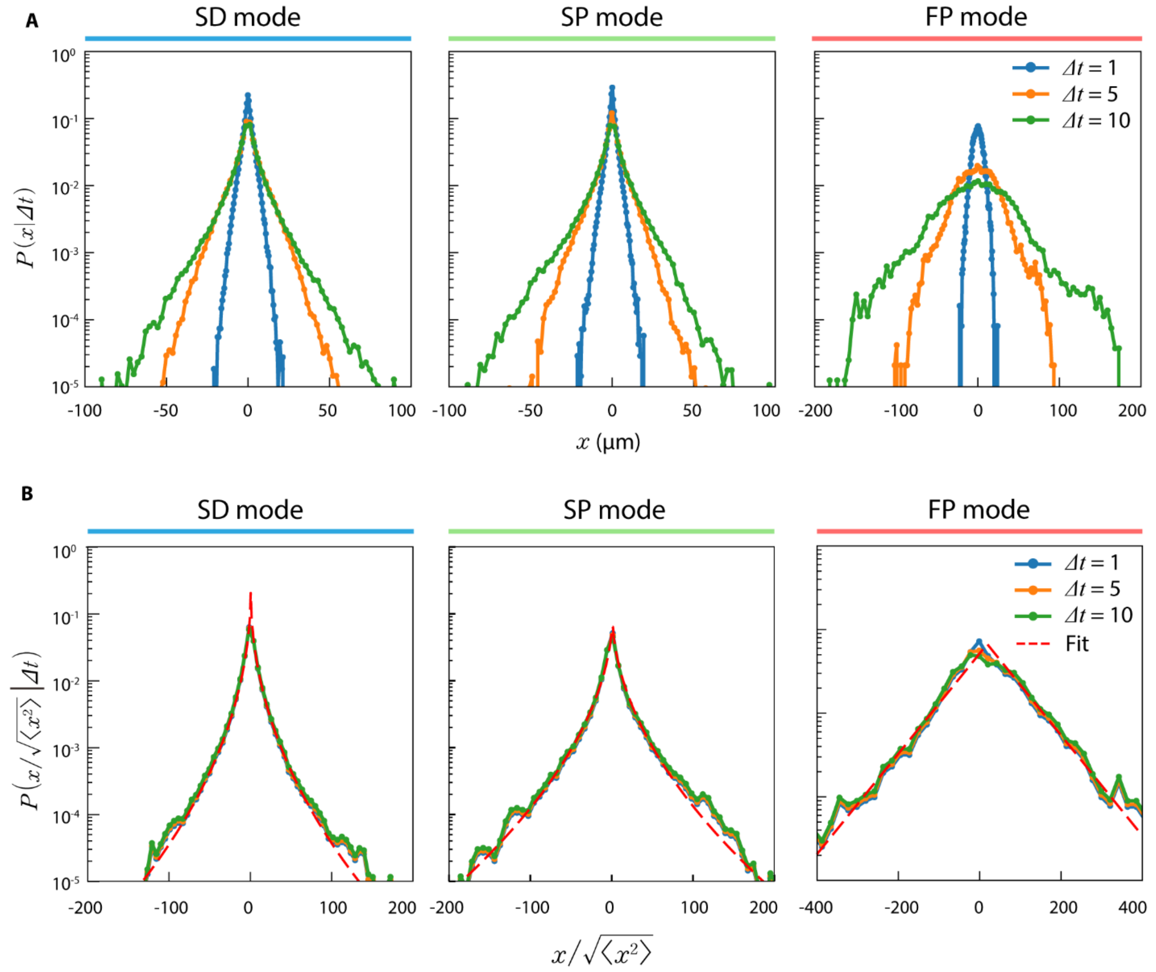

Supplementary Figure 7.

Displacement probability density functions (PDFs) $P(x|\Delta t)$ and their re-scaled PDFs. **(A)** The displayed PDFs show the x-component displacement. Similar results were obtained for y-component displacements. The three colors represent the lag time indicated in the legend of the last column. **(B)** The re-scaled PDFs were obtained by plotting the original PDFs with $\frac{\boldsymbol{x}}{\sqrt{\langle \boldsymbol{x}^2 \rangle}}$. We fitted the re-scaled PDF with a stretched-exponential function $f(x; \mu, A, B) = \mathcal{N} \exp\left[-\left(\frac{x-\mu}{A}\right)^B\right]$. The red dashed line indicates the best fit with parameters (μ, A, B): (0.48, 1.07, 0.48) (SD), (1.09, 2.84, 0.53) (SP), and (18.23, 75.26, 0.98) (FP), respectively. The fitting was performed via nonlinear least-squares minimization ("lmfit" package for Python(2)). The stretched exponent B is less than unity, indicating that DC migration is highly deviant from Gaussian dynamics ($B = 2$).

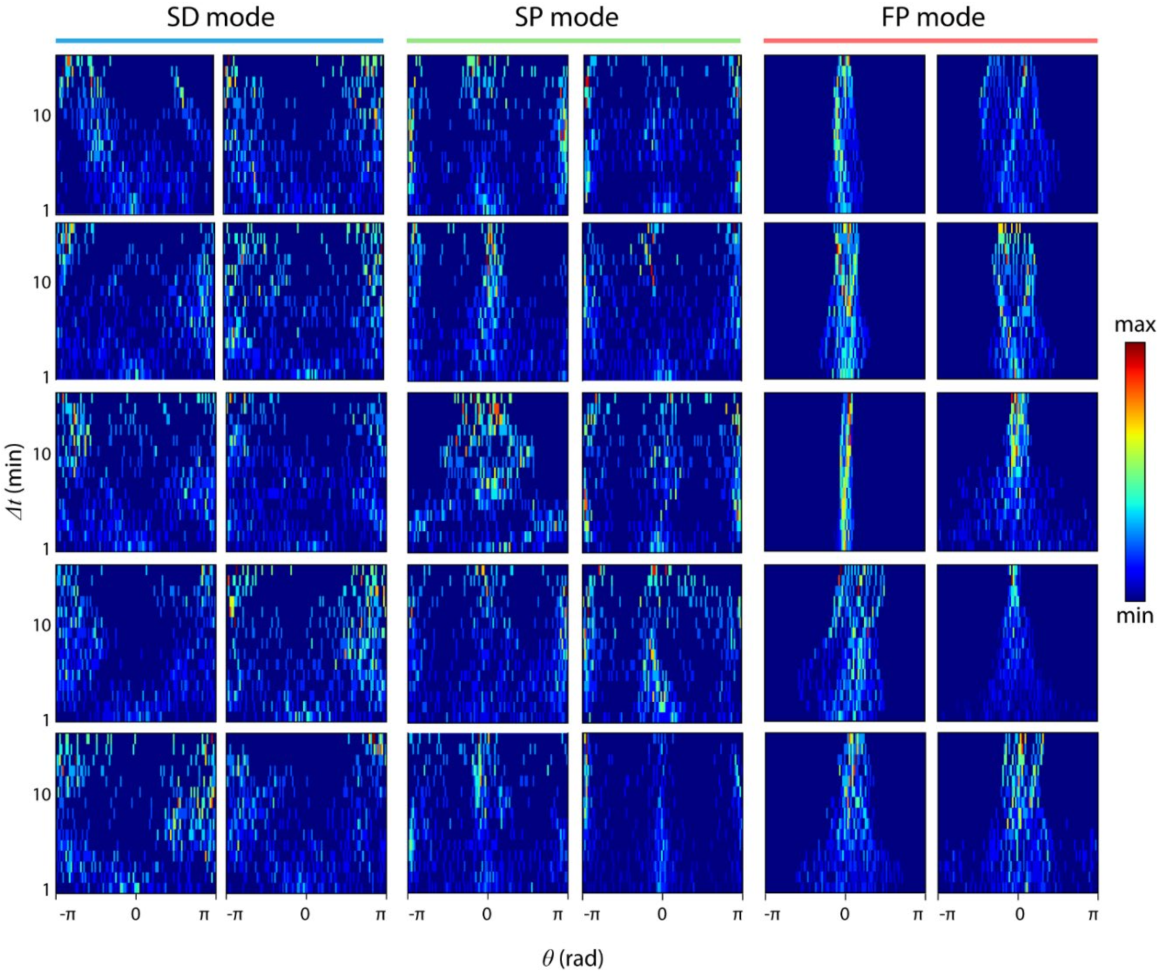

Supplementary Figure 8.
Turning angle heat maps from randomly selected individual trajectories. Ten samples were analyzed for each mode.

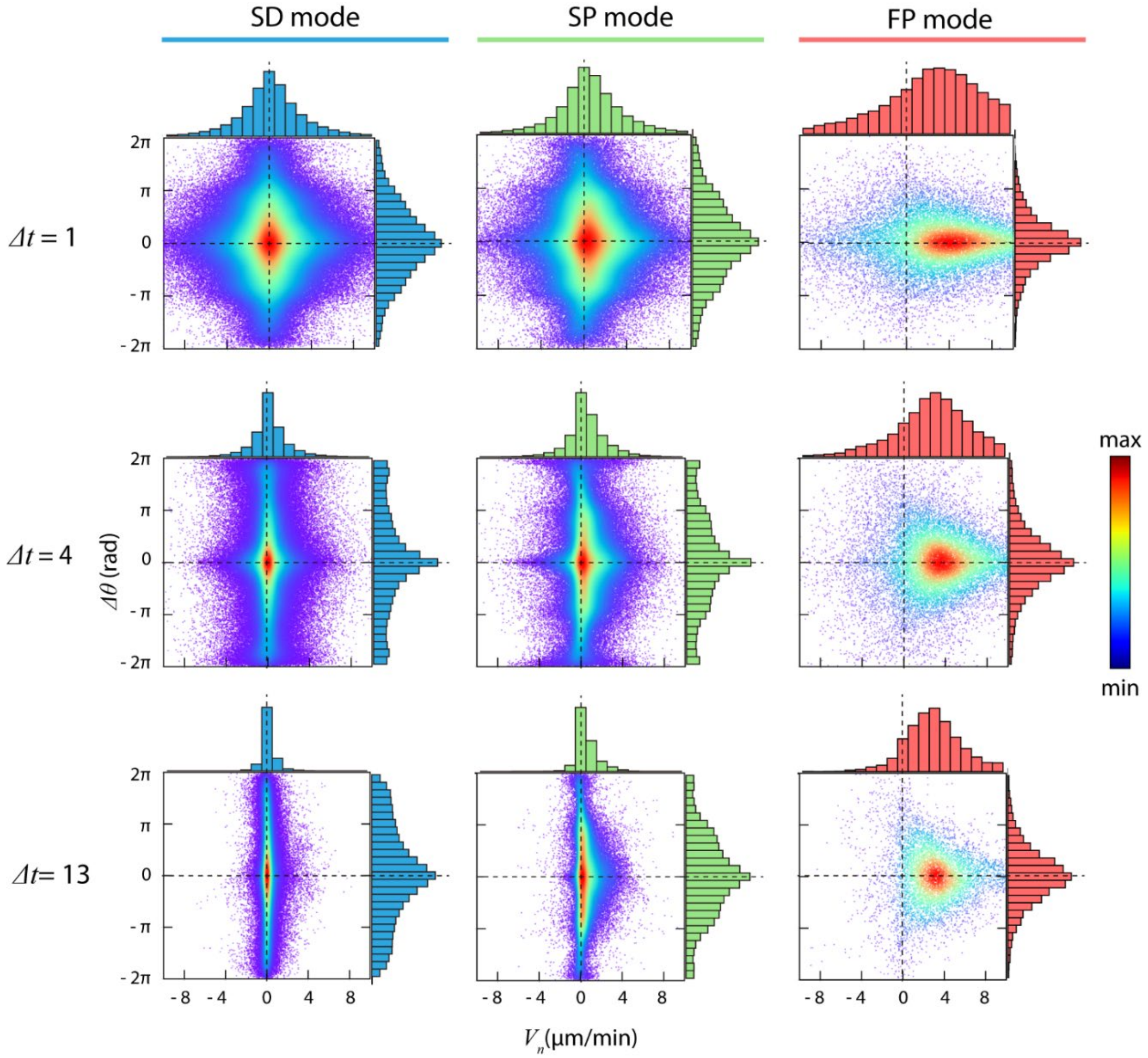

Supplementary Figure 9.

Density map of the phase-space space ($\Delta\theta_n, V_n$) for the three migration modes. Color-coded density maps at three lag times (row, min) were plotted for each mode (column). The dots indicate data from a single-track segment. Dashed lines serve as visual guides.

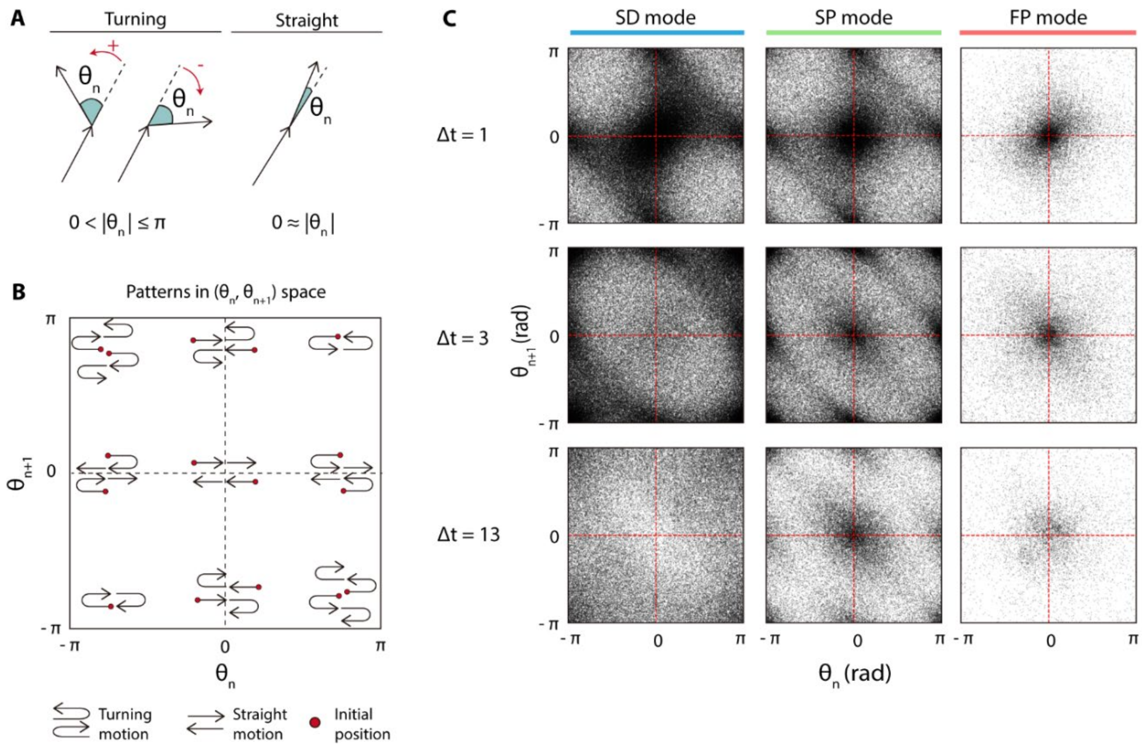

Supplementary Figure 10.
Density maps of the phase-space $(\boldsymbol{\theta_n}, \boldsymbol{\theta_{n+1}})$ for various lag times. (A) Cartoon showing turning and straight movement. The possible range of $\boldsymbol{\theta_n}$ is shown. **(B)** The nine dense spots in **(C)** and the corresponding trajectory motif. **(C)** The scatter points are collected from all trajectory samples for a given mode over the entire period. The dots indicate data from a single-track segment. Dashed lines serve as visual guides.

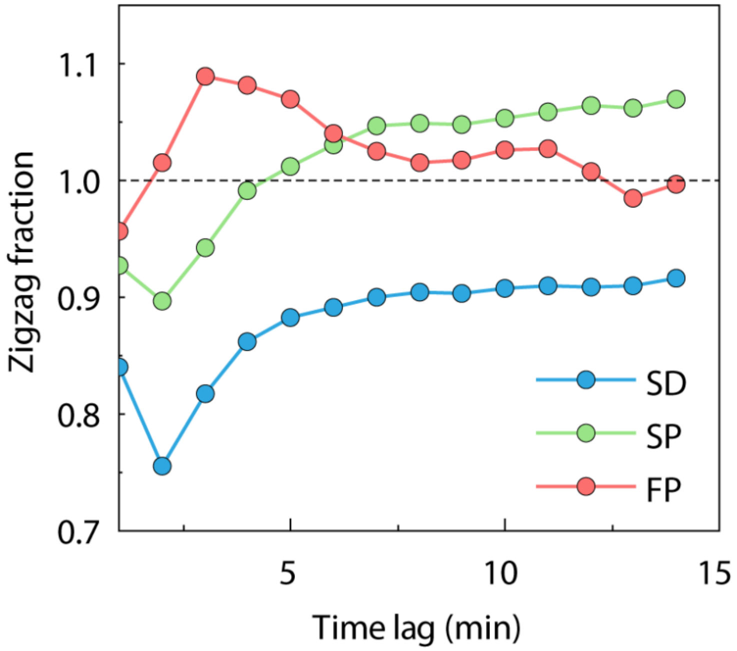

Supplementary Figure 11.
The zigzag fraction as a function of the lag time. The zigzag fraction is the ratio of the total number of scatter points between the first–third quadrant and the second–fourth quadrant in the density map. The color code represents the SD (blue), SP (green), and FP (red) modes. When the fraction becomes unity (the dashed line), it implies that the cell has the same amount of two sequential curved events over the entire migration: zigzag-like opposite turning events (left-right or right-left turn) and circular motion (left-left or right-right turn).

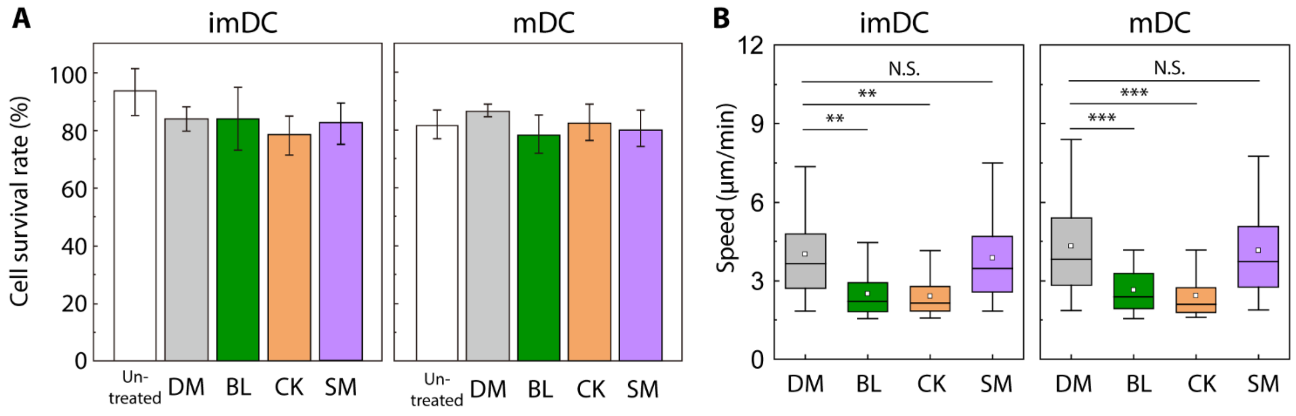

Supplementary Figure 12.

Molecular inhibition in DC migration experiments. (A) Cell survival rate of imDCs and mDCs at 24 h after drug treatments. DM: DMSO (0.1%), BL: blebbistatin (20 μM), CK: CK666 (100 μM) and SM: SMIFH2 (12.5 μM). Three independent experiments were performed. Error bar represent standard deviation. **(B)** Mean track speed of imDCs and mDCs with drug treatments. In the box plots, the bars include 95% of the points, the center corresponds to the mean, the bar correspond median and the box contains 75% of the data. Data were pooled from three independent experiments. The Kruskal–Wallis test with Dunn's *post hoc* test was used to determine statistical significance. N.S.: $P > 0.05$; **: $P < 0.01$; ***: $P < 0.001$ (imDC: DM n = 444, BL n = 213, CK n = 286, SM n = 405, mDC: DM n = 321, BL n = 102, CK n = 121, SM n=362).

Legends for Movies S1 to S12

Supplementary Movie 1.

Representative movie of imDCs migrating under gel confinement. Lines represent the last 1 h trajectories, and the color code indicates instantaneous speed. Time in hours: minutes. Background subtraction was performed to increase the contrast of the brightfield image.

Supplementary Movie 2.

Representative movie of mDCs migrating under gel confinement. Lines represent the last 1 h trajectories, and the color code indicates instantaneous speed. Time in hours: minutes. Background subtraction was performed to increase the contrast of the brightfield image.

Supplementary Movie 3.

Example of SD mode trajectory. A representative SD mode trajectory was collected in imDCs, (represented by the blue line); the line represents the last 1 h trajectory. Time in hours: minutes. Background subtraction was performed to increase the contrast of the brightfield image.

Supplementary Movie 4.

Example of SP mode trajectory. A representative SP mode trajectory was collected in imDCs (represented by the green line); the line represents the last 1 h trajectory. Time in hours: minutes. Background subtraction was performed to increase the contrast of the brightfield image.

Supplementary Movie 5.

Example of FP mode trajectory. A representative FP mode trajectory was collected in mDCs (represented by the pink line); the line represents the last 1 h trajectory. Time in hours: minutes. Background subtraction was performed to increase the contrast of the brightfield image.

Supplementary Movie 6.

imDC motility assigned to a machine-defined mode. Lines represent the last 1 h trajectories, and the color code indicates the machine-defined mode (SD: blue, SP: green, FP: pink). Time in hours: minutes. Background subtraction was performed to increase the contrast of the brightfield image.

Supplementary Movie 7.

mDC motility assigned to the machine-defined mode. Lines represent the last 1 h trajectories, and the color code indicates the machine-defined mode (SD: blue, SP: green, FP: pink). Time in hours: minutes. Background subtraction was performed to increase the contrast of the brightfield image.

Supplementary Movie 8.

imDC motility after 0.1% DMSO treatment. Lines represent the last 1 h trajectories; the color code indicates the machine-defined mode (SD: blue, SP: green, FP: pink). Time in hours: minutes. Background subtraction was performed to increase the contrast of the brightfield image.

Supplementary Movie 9.

imDC motility after 20 μM blebbistatin treatment. Lines represent the last 1 h trajectories; the color code indicates the machine-defined mode (SD: blue, SP: green, FP: pink). Time in hours: minutes. Background subtraction was performed to increase the contrast of the brightfield image.

Supplementary Movie 10.

imDC motility after 100 μM CK666 treatment. Lines represent the last 1 h trajectories; the color code indicates the machine-defined mode (SD: blue, SP: green, FP: pink). Time in hours: minutes. Background subtraction was performed to increase the contrast of the brightfield image.

Supplementary Movie 11.

imDC motility after 12.5 μM SMIFH2 treatment. Lines represent the last 1 h trajectories; the color code indicates the machine-defined mode (SD: blue, SP: green, FP: pink). Time in hours: minutes. Background subtraction was performed to increase the contrast of the brightfield image.

Supplementary Movie 12.

mDC motility after 0.1% DMSO treatment. Lines represent the last 1 h trajectories; the color code indicates the machine-defined mode (SD: blue, SP: green, FP: pink). Time in hours: minutes. Background subtraction was performed to increase the contrast of the brightfield image.

Supplementary Movie 13.

mDC motility after 20 μM blebbistatin treatment. Lines represent the last 1 h trajectories; the color code indicates the machine-defined mode (SD: blue, SP: green, FP: pink). Time in hours: minutes. Background subtraction was performed to increase the contrast of the brightfield image.

Supplementary Movie 14.

mDC motility after 100 μM CK666 treatment. Lines represent the last 1 h trajectories, and the color code indicates the machine-defined mode (SD: blue, SP: green, FP: pink). Time in hours: minutes. Background subtraction was performed to increase the contrast of the brightfield image.

Supplementary Movie 15.

mDC motility after 12.5 μM SMIFH2 treatment. Lines represent the last 1 h trajectories, and the color code indicates the machine-defined mode (SD: blue, SP: green, FP: pink). Time in hours: minutes. Background subtraction was performed to increase the contrast of the brightfield image.

Supplementary Movie 16.

Representative cyclic mode transition in imDC. Lines represent the last 1 h trajectories; the color code indicates the machine-defined mode (SD: blue, SP: green, FP: pink). Time in hours: minutes. Background subtraction was performed to increase the contrast of the brightfield image.

Reference

1. Pedregosa F, Varoquaux G, Gramfort A, Michel V, Thirion B, Grisel O, Blondel M, Prettenhofer P, Weiss R, Dubourg V, et al. Scikit-learn: Machine Learning in Python. *J Mach Learn Res* (2011) 12:2825–2830.

2. Newville M, Stensitzki T, Allen DB, Ingargiola A. LMFIT: Non-Linear Least-Square Minimization and Curve-Fitting for Python. (2014) https://doi.org/10.5281/zenodo.11813
